# Interventions in hypertension: systematic review and meta-analysis of natural and quasi-experiments

**DOI:** 10.1186/s40885-022-00198-2

**Published:** 2022-05-01

**Authors:** Tong Xia, Fan Zhao, Roch A. Nianogo

**Affiliations:** 1 Department of Epidemiology, Fielding School of Public Health, University of California, Los Angeles (UCLA), 650 Charles E. Young Drive South, Los Angeles, CA 90095 USA; 2 California Center for Population Research (CCPR), 337 Charles E. Young Drive East, Los Angeles, CA 90095 USA

**Keywords:** Hypertension, Non-randomized controlled trials as topic, Comparative effectiveness research

## Abstract

**Background:**

Hypertension is an urgent public health problem. Consistent summary from natural and quasi-experiments employed to evaluate interventions that aim at preventing or controlling hypertension is lacking in the current literature. This study aims to summarize the evidence from natural and quasi-experiments that evaluated interventions used to prevent or control hypertension.

**Methods:**

We searched PubMed, Embase and Web of Science for natural and quasi-experiments evaluating interventions used to prevent hypertension, improve blood pressure control or reduce blood pressure levels from January 2008 to November 2018. Descriptions of studies and interventions were systematically summarized, and a meta-analysis was conducted.

**Results:**

Thirty studies were identified, and all used quasi-experimental designs including a difference-in-difference, a pre-post with a control group or a propensity score matching design. Education and counseling on lifestyle modifications such as promoting physical activity (PA), promoting a healthy diet and smoking cessation consultations could help prevent hypertension in healthy people. The use of computerized clinical practice guidelines by general practitioners, education and management of hypertension, the screening for cardiovascular disease (CVD) goals and referral could help improve hypertension control in patients with hypertension. The educating and counseling on PA and diet, the monitoring of patients’ metabolic factors and chronic diseases, the combination of education on lifestyles with management of hypertension, the screening for economic risk factors, medical needs, and CVD risk factors and referral all could help reduce blood pressure. In the meta-analysis, the largest reduction in blood pressure was seen for interventions which combined education, counseling and management strategies: weighted mean difference in systolic blood pressure was − 5.34 mmHg (95% confidence interval [CI], − 7.35 to − 3.33) and in diastolic blood pressure was − 3.23 mmHg (95% CI, − 5.51 to − 0.96).

**Conclusions:**

Interventions that used education and counseling strategies; those that used management strategies; those that used combined education, counseling and management strategies and those that used screening and referral strategies were beneficial in preventing, controlling hypertension and reducing blood pressure levels. The combination of education, counseling and management strategies appeared to be the most beneficial intervention to reduce blood pressure levels.

**Supplementary Information:**

The online version contains supplementary material available at 10.1186/s40885-022-00198-2.

## Background

Cardiovascular diseases (CVD) represent the leading cause of death, accounting for one in three deaths in the United States (US) and worldwide [1–3]. One of their most potent risk factors, hypertension (also known as high blood pressure), is a common risk factor for CVD [3, 4]. Approximately 40% of adults aged 25 and over had elevated blood pressure in 2008 [3]. What is more, hypertension is responsible for at least 45% of deaths due to heart diseases and 51% of deaths due to stroke worldwide [3, 4]. In the US alone, the direct medical and indirect expenses from CVDs were estimated at approximately $329 billion in 2013 to 2014 [5]. Effective large-scale interventions to prevent or treat hypertension are therefore urgently needed to reverse this trend. Yet, as new and promising interventions are surfacing every day, the need for rigorous evaluation of these interventions to inform evidence-based policies and clinical practice is ever growing.

To this effect, several randomized clinical trials (RCT) have been conducted to evaluate interventions used to prevent hypertension or improve its control [6–8]. However, although RCTs represent the gold standard for evaluating the efficacy (i.e., impact under ideal conditions) of most health interventions because of their *high internal validity* [9, 10], they are not always feasible, appropriate or ethical for the evaluation of certain types of interventions. Furthermore, results from RCTs are not always generalizable to populations or settings of interest due to the highly selected sample and because the intervention is generally conducted under more stringent conditions (*low external validity*) [11]. To evaluate the effectiveness of an intervention (i.e., impact under real conditions) and to increase the uptake and implementation of evidence-based health interventions in the communities of interests, other types of experimental designs have been proposed. One such example is natural and quasi-experiments. The terms “natural experiments” and “quasi-experiments” are sometimes used interchangeably. In this study, and as described by others [12], we will distinguish these two concepts. Natural and quasi-experiments are similar in that, in both cases, there is no randomization of treatments or exposures (i.e., no random assignment). They differ, however, in that, natural experiments are those that involve naturally occurring or unplanned events (e.g., a national policy, new law), while quasi-experiments involve intentional or planned interventions implemented (typically for the purpose of research/evaluation) to change a specific outcome of interest (e.g., a community intervention program). Furthermore, in natural experiments, the investigator does not have control over the treatment assignment whereas in quasi-experiments, the investigator has control over the treatment assignment [12]. These experiments include difference-in-difference (DID) designs, synthetic controls and regression discontinuity designs to name a few [13–15].

As utilization of natural and quasi-experiments is increasing in public health and in the biomedical field [13–15], more natural and quasi-experiments are being conducted to evaluate interventions targeted to prevent or control hypertension [16–19]. This could be due to recent development or the reframing of classical approaches for determining causality in natural and quasi- experiments [13–15, 20]. However, unlike RCTs of interventions aiming to prevent hypertension or improve its control [6–8], consistent summary and synthesis of evidence from natural and quasi- experiments is lacking in the current literature. The primary aim of the current systematic review is to summarize the evidence from natural and quasi-experiments that have evaluated interventions used to prevent, control hypertension or reduce blood pressure levels. A secondary aim of this study is to conduct a meta-analysis to summarize intervention effectiveness.

## Methods

### Data sources and strategy

**Fig. 1:**
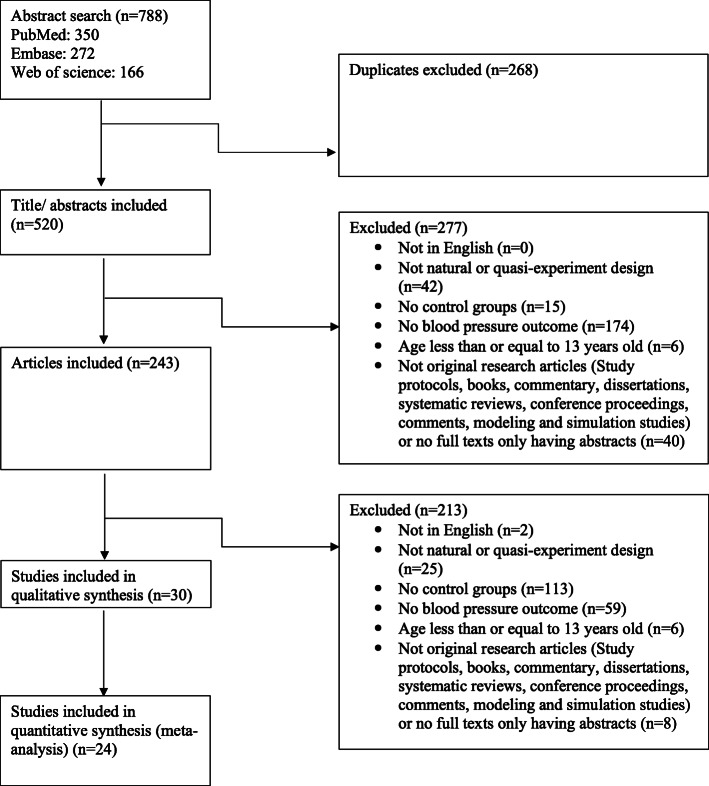
Study search and selection flow

We searched PubMed, Embase and Web of Science from January 2008 to November 2018. This time frame was selected to encompass studies that would have likely benefited from recent development and improvement in natural and quasi- experiments [13, 20]. Briefly, the search strategy consisted in intersecting keywords related to the study methods (e.g., natural experiments, quasi-experiments, DID, synthetic control, interrupted time series, etc.) with the environment or settings (e.g., community, nation, organization, etc.) and the outcome (e.g., hypertension, elevated blood pressure, etc.). The full search strategy is described in Table S1. This systematic review and meta-analysis were conducted according to the Preferred Reporting Items for Systematic reviews and Meta-Analyses (PRISMA) statement [21] (Fig. 1).

### Study selection

Two trained members (TX, FZ) screened abstracts and full-text articles. Disagreements were decided by a third member (RN). We included studies that used natural and quasi-experiments to evaluate interventions aimed at preventing hypertension, controlling hypertension or reducing blood pressure levels. The outcome measures were prevalence of hypertension and changes in mean blood pressure. Studies were excluded if they were not in English, were not a natural experiment or a quasi-experimental design, did not include a control group (as it has higher risk to internal validity due to the absence of comparison to adjust for time trends and confounding) [22], did not include blood pressure or hypertension as their outcome or included participants that were 13 years old or younger. In addition, we excluded studies that were not original research articles (e.g., study protocol, books, commentary, dissertations, conference proceedings, comments, systematic reviews, modeling and simulation studies), or had no full text available.

### Data extraction and quality assessment

The following information was extracted: study design, sample size, study duration, data source, geographic location, participants’ socio-demographic characteristics, intervention types, intervention levels (e.g., individuals, community, school, clinic and national levels as suggested by the socio-ecological model [23]), behavior targeted and outcome measures (prevalence of hypertension or mean blood pressure change) (Table 1, Table S2).

**Table 1:** Description of the study characteristics and findings among the 30 studies

First author, year	Type of study (sample size, study design, intervention duration in months, data source)	Population (geographic region, subpopulation)	Level of intervention (settings)	Intervention targeted	Reasons why the study did not use RCTs to evaluate interventions	Findings
**Studies that reported hypertension prevalence change**						
Barnidge, 2015 [24]	*• N* = 794 *•* Design: DID *•* Duration: 24 months *•* Data sources: Primary	*•* Region: America *•* Subpopulation: all genders and racial/ethnic groups *•* Participants: General population	Community	Nutrition education and give access to fruits and vegetables through community gardens **Type**: Education and counseling **Domain**: Nutrition, social and economic factors	The authors stated that a randomized design would have been hard to be conducted in community-based work.	Treatment group hypertension prevalence in the beginning: 61.0% Treatment group hypertension prevalence in the middle: 45.0%; *P*-value beginning vs. middle < 0.01 Treatment group hypertension OR beginning vs. middle: 0.52; 95% CI: (0.38; 0.71) Control group hypertension prevalence in the beginning: 46.7% Control group hypertension prevalence in the middle: 49.8%; *P*-value beginning vs. middle = 0.39 Control group hypertension OR in the beginning vs. middle: 1.11; 95% CI: (0.81; 1.54)
Sahli, 2016 [25]	*• N* = 2000 *•* Design: PPCG *•* Duration: 36 months *•* Data Source: Primary	*•* Region: Africa *•* Subpopulation: all genders and racial/ethnic groups *•* Participants: General population	Community	Healthy lifestyle promotion, education on smoking, physical activity, and diet. Free smoking cessation consultations. **Type**: Education and counseling **Domain**: Lifestyle	The authors did not justify why an RCT was not undertaken to evaluate the effectiveness of the intervention.	All participants: Treatment group hypertension prevalence in the beginning: 37.3% Treatment group hypertension prevalence at the end: 33.7%; *P*-value beginning vs. end: 0.1 Control group hypertension prevalence in the beginning: 31.1% Control group hypertension prevalence at the end: 33.4%; *P*-value beginning vs. end: 0.28 Among participants younger than 40 years old: Treatment group hypertension prevalence in the beginning: 22.8% Treatment group hypertension prevalence at the end: 16.2%; *P*-value beginning vs. end: 0.01 Control group hypertension prevalence in the beginning: 14.0% Control group hypertension prevalence at the end: 15.1%; *P*-value beginning vs. end: 0.52 Among nonobese participants: Treatment group hypertension prevalence in the beginning: 31.4% Treatment group hypertension prevalence at the end: 26.2%; *P*-value beginning vs. end: 0.03 Control group hypertension prevalence in the beginning: 21.9% Control group hypertension prevalence at the end: 25.1%; *P*-value beginning vs. end: 0.17
Comin, 2017 [18]	*• N* = 189,067 *•* Design: PPCG *•* Duration: 30 months *•* Data Source: Primary	*•* Region: Europe *•* Subpopulation: all genders and racial/ethnic groups, aged 35–74 years *•* Participants: Patients with hypertension and diabetes and hypercholesterolemia	Health center	Computerized clinical practice guidelines: General practitioners had General practitioners accessed the computerized clinical practice guidelines at least twice a day **Type**: Management **Domain**: Care	The authors did not justify why an RCT was not undertaken to evaluate the effectiveness of the intervention.	In hypertension patients: Women: Treatment group percentage of improved BP control: 9.8% Control group percentage of improved BP control: 6.7% Treatment group vs. Control group percentage of improved BP control: *P*-value < 0.001 Men: Treatment group percentage of improved BP control: 11.8% Control group percentage of improved BP control: 7.9% Treatment group vs. Control group_ percentage of improved BP control: *P*-value < 0.001
Fikri-Benbrahim, 2012 [26]	*• N* = 177 *•* Design: PPCG *•* Duration: 5 months *•* Data Source: Primary	*•* Region: Europe *•* Subpopulation: all genders and racial/ethnic groups *•* Participants: all hypertension patients	Community	Pharmacist intervention comprising (1) education about hypertension, (2) home blood pressure monitoring, and (3) referral to a physician through personalized reports when necessary **Type**: Education, counseling and management **Domain**: Lifestyle, pharmacological therapy	The authors did not justify why an RCT was not undertaken to evaluate the effectiveness of the intervention.	Treatment group percentage of controlled BP in the beginning: 71.3% Treatment group percentage of controlled BP at the end: 52.9% Treatment group percentage of controlled BP change *P*-value: 0.01 Control group percentage of controlled BP in the beginning: 55.1% Control group percentage of controlled BP at the end: 50.6% Control group percentage of controlled BP change *P*-value: 0.48 Treatment group vs. Control group percentage of controlled BP *P*-value: 0.026 Achieving BP control treatment group vs. control group OR: 2.46; 95% CI: (1.15, 5.24); *P*-value: 0.02
James, 2018 [17]	*• N* = 53,738 (12,555 with cardiovascular disease, and 41,183 with hypertension) *•* Design: DID *•* Duration: 6 months *•* Data Source: Primary	*•* Region: America *•* Subpopulation: all genders and racial/ethnic groups *•* Participants: Patients with cardiovascular disease and hypertension	Health center	Population Health Management Intervention: Adding a dedicated population health coordinator who identifies and reaches out to patients not meeting cardiovascular care goals to health management programs **Type**: Screening and referral for management **Domain**: Care	The authors did not justify why an RCT was not undertaken to evaluate the effectiveness of the intervention.	Non-Hispanic White: BP control PHC vs. non-PHC: OR = 1.13, 95% CI: (1.05, 1.22) Non-Hispanic Black: BP control PHC vs. non-PHC: OR = 1.17; 95% CI: (0.94, 1.45) Hispanic: BP control PHC vs. non-PHC: OR = 0.90; 95% CI: (0.59, 1.36) Non-Hispanic Black vs. Non-Hispanic White patients: OR = 1.05; 95% CI: (0.83, 1.31) Hispanic vs. non-Hispanic White patients: OR = 0.82; 95% CI: (0.53, 1.25)
**Studies that reported mean blood pressure change**						
***Intervention subgroup: education and counseling***						
Flannery, 2012 [27]	*• N* = 39 *•* Design: DID *•* Duration: 6 months *•* Data Source: Primary	*•* Region: America *•* Subpopulation: Only women of all racial/ethnic groups *•* Participants: nurse assistant	Organization	The Worksite Heart Health Improvement: Environmental and policy assessment; education; and ongoing motivation **Type**: Education and counseling **Domain**: Diet, physical activity, environmental and policy factors	The authors did not justify why an RCT was not undertaken to evaluate the effectiveness of the intervention.	Treatment group mean SBP in the beginning: 129.28; SD: 17.9 Treatment group mean SBP at the end: 119.88; SD: 14.76 Control group mean SBP in the beginning: 125.26; SD: 18.74 Control group mean SBP at the end: 120.3; SD: 14.43 Treatment group mean DBP in the beginning: 77.5; SD: 8.98 Treatment group mean DBP at the end: 70.84; SD: 6.82 Control group mean DBP in the beginning: 74.4; SD: 13.52 Control group mean DBP at the end: 74.43; SD: 11.77
Gemson, 2008 [28]	*• N* = 141 *•* Design: PPCG *•* Duration: 12 months *•* Data Source: Primary	*•* Region: America *•* Subpopulation: all genders and racial/ethnic groups *•* Participants: all hypertension patients	Organization	Multicomponent workplace intervention comprising informational health messages, use of a pedometer bioelectrical impedance measured body weight and physical activity education **Type**: Education and counseling **Domain**: Lifestyle, physical activity, body fat measurement	The authors did not justify why an RCT was not undertaken to evaluate the effectiveness of the intervention.	Treatment group SBP MD: −10.6; SD: 111.4 Treatment group DBP MD: −6.1; SD: 8.9 Control group SBP MD: −2.1; SD: 9.3 Control group DBP MD: 0.1; SD: 6.2
Lin, 2017 [29]	*• N* = 99 *•* Design: PPCG *•* Duration: 3 months *•* Data Source: Primary	*•* Region: Asia *•* Subpopulation: all genders and racial/ethnic groups, aged 20 years and older *•* Participants: office workers	Organization	Implementation of a “Sit Less, Walk More” Workplace intervention comprising five components: Monthly newsletters, motivational tools, pedometer challenge, environmental prompts and walking route **Type**: Education and counseling **Domain**: Lifestyle, physical activity	The authors did not justify why an RCT was not undertaken to evaluate the effectiveness of the intervention.	Treatment group SBP MD: −1.1; SD: 11.7 Treatment group DBP MD: −2.6; SD: 8.9 Control group SBP MD: 1; SD: 16.3 Control group DBP MD: 2.6; SD: 11.7
Chang, 2013 [30]	*• N* = 133 *•* Design: PPCG *•* Duration: 3 months *•* Data Source: Primary	*•* Region: Asia *•* Subpopulation: all genders and racial/ethnic groups, aged 55 years and older *•* Participants: General population	Community	60-min Tai Chi physical activity practice **Type**: Education and counseling **Domain**: Physical activity	The authors did not justify why an RCT was not undertaken to evaluate the effectiveness of the intervention.	Treatment group vs. Control group SBP: − 14.3; 95% CI: (− 19.2, − 9.4) Treatment group vs. Control group DBP: − 7.02; 95% CI: (− 10.6, − 3.4)
Verberne, 2016 [31]	*• N* = 381 *•* Design: PPCG *•* Duration: 12 months *•* Data Source: Primary and secondary	*•* Region: Europe *•* Subpopulation: all genders and racial/ethnic groups *•* Participants: overweight and obese patients	Health center	Prescription of lifestyle modifications by general practitioners which consisted of advice and referrals pertaining to diet and physical activity **Type**: Education and counseling **Domain**: Lifestyle, physical activity, nutrition	The authors did not justify why an RCT was not undertaken to evaluate the effectiveness of the intervention.	Treatment group SBP MD: − 3.5; SD: 15.4 Treatment group DBP MD: − 3.4; SD: 9 Control group SBP MD: − 3; SD: 15.5 Control group DBP MD: − 3.6; SD: 8.5
Xu, 2015 [32]	*• N =* 38 *•* Design: PPCG *•* Duration: 4 months *•* Data Source: Primary	*•* Region: America *•* Subpopulation: women of all racial/ethnic groups aged 60 years or older *•* Participants: obese patients	Community	Tai Chi physical activity and nutrition education and a behavioral weight loss program based on a modified DASH diet **Type**: Education and counseling **Domain**: Lifestyle, physical activity, nutrition	The authors stated that although the study of the intervention has been done as an RCT in a clinic, they wanted to translate the intervention in a community setting.	Treatment group vs. Control group SBP: − 8.9; 95% CI: (− 19.1, 1.4) Treatment group vs. Control group DBP: − 3.4; 95% CI: (− 9.8, 3.09)
Zhu, 2018 [33]	*• N* = 36 *•* Design: PPCG *•* Duration: 4 months *•* Data Source: Primary	*•* Region: America *•* Subpopulation: all genders and racial/ethnic groups aged 18–25 years old *•* Participants: office workers	Organization	A workplace physical activity intervention comprising sit-stand workstations and sitting-specific motivational support and instructions **Type**: Education and counseling **Domain**: Physical activity	The authors stated that a randomized design would have been hard to be conducted in real world organizational settings.	Treatment group mean SBP in the beginning: 119.1; SD: 16.4 Treatment group mean SBP at the end: 121.4; SD: 19.8 Control group mean SBP in the beginning: 118.8; SD: 12.2 Control group mean SBP at the end: 123.8; SD: 10.6 Treatment group mean DBP in the beginning: 75.6; SD: 10.3 Treatment group mean DBP at the end: 77.2; SD: 12.2 Control group mean DBP in the beginning: 77.2; SD: 10.8 Control group mean DBP at the end: 78.9; SD: 6.9
Kamran, 2016 [34]	*• N* = 138 *•* Design: PPCG *•* Duration: 6 months *•* Data source: Primary	*•* Region: Asia *•* Subpopulation: all genders and racial/ethnic groups *•* Participants: Patients with hypertension	Health center	Nutritional advice/education about the DASH approach which was presented in group teaching sessions **Type**: Education and counseling **Domain**: Diet	The authors did not justify why an RCT was not undertaken to evaluate the effectiveness of the intervention.	Treatment group SBP MD: − 13.0; SD: 10.2 Treatment group DBP MD: − 7.3; SD: 5.3 Control group SBP MD: 0.5; SD: 12.2 Control group DBP MD: − 0.7; SD: 7.8
Ibrahim, 2016 [35]	*• N* = 268 *•* Design: PPCG *•* Duration: 12 months *•* Data source: Primary	*•* Region: Asia *•* Subpopulation: all genders and racial/ethnic groups aged between 18 and 65 years old *•* Participants: Patients with prediabetes	Community	Group-based sessions and individual counseling to reinforce behavioral change (diet, physical activity) **Type**: Education and counseling **Domain**: Lifestyle	The authors stated that a randomized design would have been hard to be conducted in community-based work.	Treatment group vs. Control group SBP: − 1.71; 95% CI: (− 3.97, 0.56) Treatment group vs. Control group DBP: − 2.63; 95% CI: (− 3.79, − 1.48)
Kassim, 2017 [36]	*• N* = 328 *•* Design: PPCG *•* Duration: 6 months *•* Data source: Primary	*•* Region: Asia *•* Subpopulation: low socio-economic status housewives aged 18–59 years old, all ethnic groups *•* Participants: Overweight and obese housewives	Community	Lifestyle interventions consisting of a healthy diet, physical activity, and self-monitoring behaviors **Type**: Education and counseling **Domain**: Lifestyle	The authors stated that a randomized design would have been hard to be conducted in community-based work.	Treatment group mean SBP in the beginning: 122.29; SD: 16.84 Treatment group mean SBP at the end: 116.45; SD: 14.62 Control group mean SBP in the beginning: 120.63; SD: 14.62 Control group mean SBP at the end: 114.59; SD: 14.86 Treatment group mean DBP in the beginning: 78.59; SD: 12.03 Treatment group mean DBP at the end: 77.14; SD: 11.15 Control group mean DBP in the beginning: 77.83; SD: 9.54 Control group mean DBP at the end: 76.10; SD: 9.49
Fazliana, 2018 [37]	*• N =* 328 *•* Design: PPCG *•* Duration: 12 months *•* Data Source: Primary	*•* Region: Asia *•* Subpopulation: housewives aged 18–59 years old *•* Participants: Overweight and obese housewives	Community	The weight loss intervention, consisted of individual diet counseling, group exercise and self-monitoring tools **Type**: Education and counseling **Domain**: Lifestyle	The authors did not justify why an RCT was not undertaken to evaluate the effectiveness of the intervention.	Treatment group SBP MD in the 6 months: − 6.81; 95% CI: (− 9.72, − 3.90) Treatment group DBP MD in the 6 months: − 1.71; 95% CI: (− 3.71, 0.28) Control group SBP MD in the 6 months: − 7.95; 95% CI: (− 11.69, − 4.20) Control group DBP MD in the 6 months: − 1.73; 95% CI: (− 4.12, 0.67)
Sahli, 2016 [25]	*• N =* 2000 *•* Design: PPCG *•* Duration: 36 months *•* Data Source: Primary	*•* Region: Africa *•* Subpopulation: all genders and racial/ethnic groups *•* Participants: General population	Community	Healthy lifestyle promotion, education on smoking, physical activity, and diet. **Type**: Education and counseling **Domain**: Lifestyle	The authors did not justify why an RCT was not undertaken to evaluate the effectiveness of the intervention.	Treatment group mean SBP in the beginning: 132.4; SD: 19.2 Treatment group mean SBP at the end: 130.6; SD: 17.7 Control group mean SBP in the beginning: 129.7; SD: 17.8 Control group mean SBP at the end: 130.4; SD: 17.9 Treatment group mean DBP in the beginning: 78.7; SD: 11.7 Treatment group mean DBP at the end: 76.9; SD: 11.1 Control group mean DBP in the beginning: 78.1; SD: 10.8 Control group mean DBP at the end: 76.7; SD: 11.0
***Intervention subgroup: management***						
Panattoni, 2017 [19]	*• N* = 11,190 (hypertension patients aged 18–59 years: *N* = 4385; hypertension patients aged 60–80 years: *N* = 4620;diabetes patients aged 18–75 years: *N* = 3768) *•* Design: DID *•* Duration:12 months *•* Data Source: Secondary	*•* Region: America *•* Subpopulation: all genders and racial/ethnic groups *•* Participants: Patients with hypertension or diabetes	Health center	Team based chronic care model, redesigned primary care visits to enhance the self-management support provided by physicians, and a health coaching program. **Type**: Management **Domain**: Care	The authors did not justify why an RCT was not undertaken to evaluate the effectiveness of the intervention.	Adjusted results: Among diabetes patients aged 18–75 years over the 6-month period: Treatment group vs. Control group SBP: − 1.65; 95% CI: (− 3.68, 0.39) Treatment group vs. Control group DBP: − 1.13; 95% CI: (− 2.23, − 0.04) Among hypertension patients aged 18–59 years over the first 6-month period: Treatment group vs. Control group SBP: − 0.75; 95% CI: (− 2.82, 1.31) Treatment group vs. Control group DBP: − 0.58; 95% CI: (− 1.87, 0.71) Among hypertension patients aged 60–80 years over the 6-month period: Treatment group vs. Control group SBP: − 0.96; 95% CI: (− 2.86, 0.95) Treatment group vs. Control group DBP: − 1.03; 95% CI: (− 2.07, 0.01) Unadjusted results of diabetes aged 18–75 years: Treatment group mean SBP in the beginning: 126.5; SD: 12.7 Treatment group mean SBP at the end: 125.5; SD: 15.3 Control group mean SBP in the beginning: 129.8; SD: 13.2 Control group mean SBP at the end: 129.8; SD: 15.7 Treatment group mean DBP in the beginning: 76.4; SD: 7.7 Treatment group mean DBP at the end: 74.4; SD: 8.8 Control group mean DBP in the beginning: 76.2; SD: 7.9 Control group mean DBP at the end: 74.9; SD: 9.4
Miao, 2018 [38]	*• N* = 1673 pairs *•* Design: PSM & DID *•* Duration: 12 months *•* Data Source: Primary	*•* Region: Asia *•* Subpopulation: all genders and racial/ethnic groups *•* Participants: Patients with hypertension	Community	Improve the performance of social health insurance system through increasing outpatient expenditure reimbursement ratio. **Type**: Management **Domain**: Payment	The authors did not justify why an RCT was not undertaken to evaluate the effectiveness of the intervention.	SBP MD: −2.9, *P*-value = 0.011 DBP MD: − 7.9,*P*-value = 0.508
Scanlon, 2008 [39]	*• N* = 2067 *•* Design: DID & PSM *•* Duration: 12 months *•* Data source: Secondary	*•* Region: America *•* Subpopulation: all genders and racial/ethnic groups *•* Participants: Patients with diabetes	Health center	Team-Based Treatment: Collaborative team-based treatment with teams comprising a physician or nurse practitioner, care manager, medical assistant, information specialist, and a part-time social worker **Type**: Management **Domain**: Care	The authors did not justify why an RCT was not undertaken to evaluate the effectiveness of the intervention.	All CareSouth patients: SBP MD per year: − 0.88;*P*-value: 0.01 CareSouth patients with baseline SBP > 140: SBP MD per year: − 2.2;*P*-value: 0.04); 95% CI: (− 3.88, − 0.44)
***Intervention subgroup: education, counseling and management***						
Darviri, 2016 [40]	*• N* = 548 *•* Design: DID *•* Duration: 2 months *•* Data Source: Primary	*•* Region: Europe *•* Subpopulation: all genders and racial/ethnic groups, aged 18–65 years, residents of Athens and literate in Greek *•* Participants: all hypertension and pre-hypertension patients	Nation	Stress management: Biofeedback-assisted diaphragmatic breathing and relaxation, lifestyle counseling, cognitive reconstruction and other relaxation techniques **Type**: Education, counseling and management **Domain**: Stress	The authors did not justify why an RCT was not undertaken to evaluate the effectiveness of the intervention.	SBP MD: − 2.62; 95% CI: (− 3.96, − 1.29) DBP MD: − 1; 95% CI: (− 1.9, − 0.93)
Fernandez, 2008 [41]	*• N* = 65 *•* Design: PPCG *•* Duration: 4 months *•* Data Source: Primary	*•* Region: America *•* Subpopulation: all genders and Black, African American, Latino or Hispanic racial/ethnic groups, aged 60 and older *•* Participants: all hypertension patients	Community	Lifestyle modification education about hypertension, antihypertensive medications, diet and physical activity, and adherence to medication **Type**: Education, counseling and management **Domain**: Pharmacological therapy, diet and physical activity	The authors did not justify why an RCT was not undertaken to evaluate the effectiveness of the intervention.	Treatment group SBP MD: − 13; SD: 18.5 Treatment group DBP MD: − 5.6; SD: 10.8 Control group SBP MD: − 10.6; SD: 24 Control group DBP MD: − 3; SD: 11.8
Fikri-Benbrahim, 2012 [26]	*• N =* 177 *•* Design: PPCG *•* Duration: 5 months *•* Data Source: Primary	*•* Region: Europe *•* Subpopulation: all genders and racial/ethnic groups *•* Participants: all hypertension patients	Community	Pharmacist intervention comprising (1) education about hypertension, (2) home blood pressure monitoring, and (3) referral to a physician through personalized reports when necessary **Type**: Education, counseling and management **Domain**: Lifestyle. Pharmacological therapy	The authors did not justify why an RCT was not undertaken to evaluate the effectiveness of the intervention.	Treatment group SBP MD: − 6.8; SD: 13.7 Treatment group DBP MD: − 2.1; SD: 8.9 Control group SBP MD: − 2.1; SD: 9.3 Control group DBP MD: 0.1; SD: 6.2
Jung, 2017 [42]	*• N* = 64 *•* Design: PPCG *•* Duration: 7 months *•* Data Source: Primary	*•* Region: Asia *•* Subpopulation: all genders and racial/ethnic groups, aged 65 years or older *•* Participants: all hypertension patients	Community	In-class educational on hypertension management, community-based eHealth monitoring, and monthly telephone counseling **Type**: Education, counseling and management **Domain**: Lifestyle	The authors stated that a randomized design would have been hard to be conducted in community-based work.	Treatment group SBP MD: − 11.4; SD: 12.5 Treatment group DBP MD: − 3; SD: 8.5 Control group SBP MD: − 0.6; SD: 11.7 Control group DBP MD: 0.6; SD: 9.5
Hussain, 2016 [43]	*• N* = 629 *•* Design: DID & PSM *•* Duration: 3 months *•* Data sources:Primary and secondary	*•* Region: America *•* Subpopulation: all genders and racial/ethnic groups, aged 40–74 years *•* Participants: all hypertension patients	Health center	Nutritional and pharmacological therapy and lifestyle counseling, and medication adherence **Type**: Education, counseling and management **Domain**: Lifestyle, pharmacological therapy	The authors stated that a randomized design would have been hard to be conducted in a pragmatic clinical setting.	SBP MD: 9;*P*-value < 0.001 DBP MD: 4; *P*-value: 0.004
Miao, 2016 [44]	*• N* = 1426 *•* Design: DID *•* Duration: 24 months *•* Data Source: Primary	*•* Region: Asia *•* Subpopulation: all genders and racial/ethnic groups *•* Participants: Patients with hypertension	Health center	Integration of preventive-curative services delivery and cooperation among village-town-county physicians, including educating on smoking cessation, moderate drinking, light and healthy diet, regular exercise and to take blood pressure drugs regularly, monitor the blood pressure **Type:** Education, counseling and management **Domain:** Lifestyle, pharmacological therapy	The authors did not justify why an RCT was not undertaken to evaluate the effectiveness of the intervention.	SBP MD: − 5.62; SD: 16.49 DBP MD: − 5.43; SD: 15.03
Visanuyothin, 2018 [45]	*• N* = 128 *•* Design: PPCG *•* Duration: 5 months *•* Data source: Primary	*•* Region: Asia *•* Subpopulation: all genders and racial/ethnic groups *•* Participants: Patients with hypertension	Health center	Integrated program with home blood pressure monitoring and village health volunteers. Group-based health education on home blood pressure monitoring and self-monitoring during workshops, including hypertension measurement skills, self-management **Type**: Education, counseling and management **Domain**: Care	The authors stated that a randomized design would have been hard to be conducted in a pragmatic clinical setting.	Treatment group mean SBP in the beginning: 134.72; SD: 13.38 Treatment group mean SBP at the end: 130.21; SD: 11.88 Control group mean SBP in the beginning: 129.27; SD: 14.01 Control group mean SBP at the end: 131.89; SD: 12.31 Treatment group mean DBP in the beginning: 80.66; SD: 8.22 Treatment group mean DBP at the end: 77.59; SD: 7.94 Control group mean DBP in the beginning: 75.70; SD: 7.50 Control group mean DBP at the end: 77.29; SD: 6.82
***Intervention subgroup: screening and referral for management***						
Berkowitz, 2017 [16]	*• N* = 5125 *•* Design: DID *•* Duration: 31 months *•* Data source: Primary	*•* Region: America *•* Subpopulation: all genders and racial/ethnic groups *•* Participants: All patients.	Health center	Addressing unmet basic resource needs: Screening for unmet needs at clinic visits, and offering those who screen positive to meet with an advocate to help obtain resources, or receive brief information provision **Type**: Screening and referral for management **Domain**: Social and economic risk factors	The authors stated that a randomized design would have been hard to be conducted in a pragmatic clinical setting. The findings are more generalizable to other primary care settings than using RCTs.	SBP MD: − 2.6; 95% CI: (−3.5, − 1.7) DBP MD: − 1.4; 95% CI: (− 1.9, − 0.9)
Scharf, 2016 [46]	*• N* = 791 *•* Design: DID *•* Duration: 24 months *•* Data Source: Primary	*•* Region: America *•* Subpopulation: all genders and racial/ethnic groups aged 18 years and older *•* Participants: patients with serious mental illness	Health center	Primary and Behavioral Health Care Integration program: Screening and referral for general medical illness prevention and treatment, registry and tracking systems for general medical needs and outcomes, care management, and prevention and wellness services **Type**: Screening and referral for management **Domain**: Care	The authors did not justify why an RCT was not undertaken to evaluate the effectiveness of the intervention.	Treatment group SBP MD: − 14; SE: 1 Treatment group DBP MD: − 13; SE: 1 Control group SBP MD: − 13; SE: 2 Control group DBP MD: − 10; SE: 1
Chang, 2016 [47]	*• N* = 138,788 *•* Design: DID *•* Duration: 24 months *•* Data Source: Primary and secondary	*•* Region: Europe *•* Subpopulation: all genders and racial/ethnic groups, aged 40–74 years • Participants: All patients	Nation	Participated in The National (England) Health Service Check—a Cardiovascular risk assessment and management program: screening, tailored management strategies including lifestyle advice **Type**: Screening and referral for management **Domain**: CVD risk factors	The authors did not justify why an RCT was not undertaken to evaluate the effectiveness of the intervention.	SBP MD: − 2.51; 95% CI: (− 2.77, − 2.25) DBP MD: − 1.46; 95% CI: (− 1.62, − 1.29)
Yu, 2017 [48]	*• N* = 10,262 *•* Design: PSM *•* Duration: 12 months *•* Data Source: Primary	*•* Region: Asia *•* Subpopulation: all genders and racial/ethnic groups, aged less than 80 years *•* Participants: all hypertension patients	Health center	Risk assessment and management program for patients with hypertension in public primary care clinics: Standardized CVD-risk assessment, hypertensive complication screening as well as adherence to medications and lifestyles **Type**: Screening and referral for management **Domain**: Lifestyle	The authors stated that a randomized design would have been hard to be conducted in a pragmatic clinical setting.	Treatment group mean SBP in the beginning: 148.7; SD: 8.18 Treatment group mean SBP at the end: 136.85; SD: 9.64 Control group mean SBP in the beginning: 148.68; SD: 8.34 Control group mean SBP at the end: 137.68; SD: 10.48 Treatment group mean DBP in the beginning: 81.7; SD: 9.34 Treatment group mean DBP at the end: 77.58; SD: 8.37 Control group mean DBP in the beginning: 81.74; SD: 9.09 Control group mean DBP at the end: 77.6; SD: 8.56
van de Vijver, 2016 [49]	*• N* = 2764 *•* Design: DID *•* Duration: 18 months *•* Data Source: Primary	*•* Region: Africa *•* Subpopulation: all genders and racial/ethnic groups *•* Participants: Patients with hypertension	Community	Awareness campaigns, household visits for screening, referral and treatment, promoting long-term retention in care: **Type**: Screening and referral for management **Domain**: Care	The authors did not justify why an RCT was not undertaken to evaluate the effectiveness of the intervention.	SBP MD: − 0.32; 95% CI: (− 2.48, 1.83) DBP MD: 1.09; 95% CI: (− 0.29, 2.46)

RCT, randomized clinical trial; DID, difference-in-difference; Treatment group, intervention or treatment group; OR, odds ratio; CI, confidence interval; PPCG, pre-post with a control group; BP, blood pressure; PHC, population health coordinator; SBP, systolic blood pressure; SD, standard deviation; DBP, diastolic blood pressure; MD, mean difference; DASH, Dietary Approaches to Stop Hypertension; PSM, propensity score matching; SE, standard error; CVD, cardiovascular disease

The interventions were classified by strategies into four types:

1. Education and counseling: This subcategory includes strategies that aim at educating and providing knowledge and counseling to participants on lifestyle modifications (e.g., increasing physical activity (PA), eating better, avoiding or stopping smoking, etc.).
2. Management: This subcategory includes strategies that aim at monitoring patients’ metabolic factors and chronic diseases (e.g., blood pressure, cholesterol level, etc.) as well as patients’ adherence to medication. These strategies are generally done or facilitated by physicians, general practitioners (e.g., by assessing computerized clinical guidelines in the electronic health record management system), nurses, other staffs, or patients themselves.
3. Education, counseling and management: This subcategory combines education and counseling strategies with management strategies as described above.
4. Screening and referral for management: This subcategory includes strategies that aim at screening for (i.e., checking for the presence of) economic risk factors, medical needs, and CVD risk factors, followed by the referral of participants who screened positive to professionals who specialize in the management of those needs.

We also classified the interventions by settings into (1) community level; (2) health center level (i.e., primary care center or general practices), (3) organization level and (4) nationwide. In addition, we have classified the intervention by duration of the study into short-term (i.e., participants were followed for less than 12 months) and long-term (i.e., participants were followed for longer than or equal to 12 months).

We implemented the Cochrane Risk of Bias Tool for risk of bias and used the Grading of Recommendations, Assessment, Development, and Evaluation (GRADE) approach to assess the quality of the evidence for mean blood pressure change outcome [50], since the meta-analysis focused on this outcome. The risk of bias for studies included in this review could be found in Table S3 and the quality of studies has also been summarized in Table S4.

### Meta-analysis

To summarize the effectiveness of interventions on mean blood pressure changes, we also conducted a meta-analysis. Due to the high heterogeneity in the studies and interventions, we undertook a random-effects model and only summarized the effectiveness of intervention strategies by subgroup defined by intervention types, settings and duration. We estimated the weighted mean difference (WMD) of blood pressure and 95% confidence intervals (CIs). The studies included in the meta-analysis were only those whose outcomes were mean differences (MDs) in blood pressure (*n* = 27) [16, 19, 25–49] as these studies provided the data needed for performing the meta-analysis. Three studies [38, 39, 43] were excluded as they did not provide enough information to compute the standard errors (SEs). To estimate the average effect of the intervention when not directly provided, we subtracted the before-and-after change in the intervention group from that in the control group or subtracted the intervention-to-control difference at follow-up to that at baseline (pre-post design with a control group). Methods to calculate intervention impact and SEs were outlined in the appendix (Figs. S1, S2, Table S5).

**Fig. 2:**
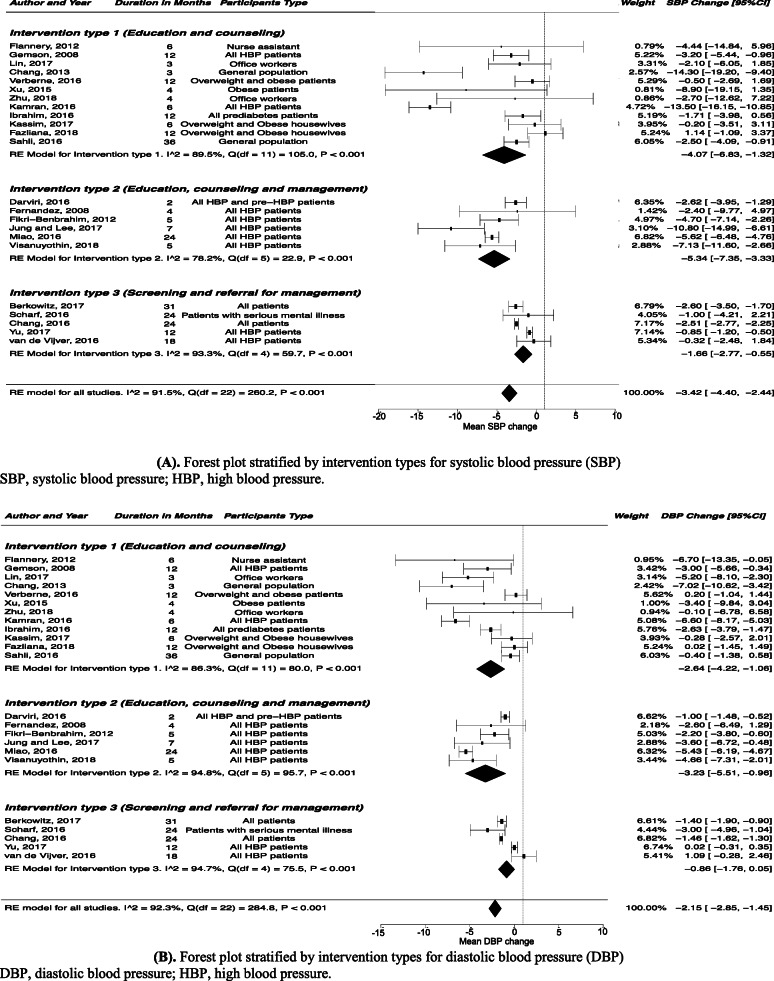
Forest plot stratified by intervention types for blood pressure. **A** Forest plot stratified by intervention types for systolic blood pressure (SBP). **B** Forest plot stratified by intervention types for diastolic blood pressure (DBP)

We presented the meta-analysis results using forest plots (Table 2, Fig. 2, Figs. S3, S4). We assessed the heterogeneity by using the *I*^*2*^ (Table 2, Fig. 2, Figs. S3, S4). We did not perform meta-regression as it is not recommended when the number of studies is small (< 10 studies per covariate) [51]. We assessed publication bias by using funnel plots of SEs (Figs. S5, S6, S7). To test the robustness of our results, we performed sensitivity analyses by removing one study at a time from the pool of studies to assess its impact on the findings (Tables S6 , S7, S8, Figs. S8, S9, S10). Data were analyzed with Stata 15.1 (StataCorp LLC, College Station, TX, USA).

**Table 2:** Summary estimates of the subgroup meta-analysis

	SBP			DBP		
	No. of Studies	Effect size, mean change, mmHg (95% CI)	***I***^***2***^, %	No. of Studies	Effect size, mean change, mmHg (95% CI)	***I***^***2***^, %
**Subgroup meta-analysis by intervention type**						
Education and counseling	12	−4.07 (− 6.83, − 1.32)	89.5	12	−2.64 (− 4.22, − 1.06)	86.3
Education, counseling and management	6	−5.34 (−7.35, − 3.33)	78.2	6	−3.23 (− 5.51, − 0.96)	94.8
Screening and referral for management	5	− 1.66 (− 2.77, − 0.55)	93.3	5	−0.86 (− 1.76, 0.05)	94.7
**Subgroup meta-analysis by intervention setting**						
Community	10	−3.77 (− 6.17, − 1.37)	84.6	10	−1.58 (− 2.79, − 0.36)	75.6
Health center	8	− 3.77 (− 5.78–1.76)	96.3	8	−2.57 (− 4.07, − 1.06)	97.0
Nation	2	− 2.51 (− 2.77, − 2.26)	0.0	2	−1.29 (− 1.72, − 0.85)	67.7
Organization	4	−2.97 (− 4.86, − 1.09)	0.0	4	−3.92 (− 5.80, − 2.04)	4.3
**Subgroup meta-analysis by intervention duration**						
Short duration	12	− 6.25 (− 9.28, − 3.21)	87.1	12	− 3.54 (− 5.21, − 1.87)	84.3
Long duration	12	−1.89 (− 2.80, − 0.97)	92.3	12	− 1.33 (− 2.11, − 0.55)	94.9

SBP, systolic blood pressure; DBP, diastolic blood pressure; CI, confidence interval

## Results

Overall, 788 titles of potentially relevant studies were identified and screened. In total, 545 were excluded and 243 full papers were retrieved, then 30 studies were included in the final sample **(**Fig. 1**)**.

### Study characteristics

Of the 30 studies included in this review [16–19, 24–49], three studies reported changes in hypertension prevalence, among which one study reported preventing hypertension in the general population [24] and two studies reported blood pressure control in patients with hypertension [17, 18]; 25 studies reported mean blood pressure changes [16, 19, 27–49]; two studies reported both outcome measures (changes in hypertension prevalence and mean blood pressure changes) [25, 26]. Thirteen studies used education and counseling intervention strategies [24, 25, 27–37]; four studies used management intervention strategies [18, 19, 38, 39]; seven studies combined education, counseling and management intervention strategies [26, 40–45]; and six studies used screening and referral for management intervention strategies [16, 17, 46–49]. Fourteen studies followed participants for less than 12 months (i.e., short-term interventions) [17, 26, 27, 29, 30, 32–34, 36, 40–43, 45]. Twelve studies were conducted in the US [16, 17, 19, 24, 27, 28, 32, 33, 39, 41, 43, 46] and most studies included both genders [16–19, 24–26, 28–31, 33–49] and all racial/ethnic groups [16–19, 24–40, 42–49]. We found no natural experiments according to the definition used in this study (Table 1, Table S2).

### Quality ratings

According to the Cochrane Risk of Bias Tool, most studies included in this review were found to have a high risk of bias **(**Table S3). This was so because the Cochrane Risk of Bias Tool was mostly designed for RCTs. Studies included in this review only used quasi-experiment designs and as such did not use randomization, allocation concealment, blinding of participants and personnel, and blinding of outcome assessment. Using the GRADE approach, the quality of evidence was deemed of low quality for the mean systolic blood pressure (SBP) and diastolic blood pressure (DBP) change outcome (Table S4).

### Studies that reported prevalence of hypertension in the general population or changes in the prevalence of controlled blood pressure in hypertension patients after intervention

#### Outcome of interest: prevention of hypertension in healthy people

##### Education and counseling intervention strategies

Two studies evaluated the education and counseling intervention strategies, and both found that those strategies could help prevent hypertension in healthy people [24, 25]. One study in the US found that nutritional education and giving access to fruits and vegetables through community gardens helped reduce hypertension prevalence (61.0% vs. 45.0%; *P* < 0.01), whereas the prevalence of hypertension in the control group did not change (46.7% vs. 49.8%; *P* = 0.39) [24]. The other study in Africa showed that an education strategy which promoted PA and healthy diet and combined with free smoking cessation consultations could help reduce the prevalence of hypertension (22.8% vs. 16.2%; *P* = 0.01), compared to that in control group (14.0% vs. 15.1%; *P* = 0.52) [25].

#### Outcome of interest: improvement of hypertension control in patients with hypertension

##### Management intervention strategies

A study in the US showed that patients whose general practitioners accessed the computerized clinical practice guideline at least twice a day improved their hypertension control compared to the patients whose general practitioners never accessed the computerized clinical practice guideline (*P* < 0.001) [18].

##### Education, counseling and management intervention strategies

A study in the US found that patients who received education about hypertension and did home blood pressure monitoring had a better control of their hypertension compared to the control group (*P* = 0.03) [26].

##### Screening and referral for management intervention strategies

A study in the US showed that for White patients, interventions which involved a coordinator who identified and reached out to patients not meeting CVD goals and linked them to management programs could improve the odds of blood pressure control (odds ratio, 1.13; 95% CI, 1.05 to 1.22) compared to no intervention [17].

### Studies that reported mean blood pressure changes after intervention

#### Outcome of interest: reduction in mean blood pressure

##### Education and counseling intervention strategies

Seven [25, 27–30, 34, 35] of twelve [25, 27–37] (58.3%) studies showed that the education and counseling intervention strategies could help reduce mean blood pressure compared to the control group. Education and counseling interventions targeting lifestyle modifications (e.g., diet and PA) have been found effective in reducing blood pressure in the workplace. A study in US female nursing assistants found that combining education and continuing motivation (e.g., counseling on questions of interventions and receiving feedback) on diet and PA led to more reduction in DBP compared to the control group who only received the education (MD, − 6.70 mmHg; 95% CI, − 13.35 to − 0.05) [27]. Two other studies also found that multi-component lifestyle interventions in the workplace including sharing health information by messages, putting up posters, using pedometers, and giving education on PA could help healthy employees or employees with hypertension lower blood pressure [28, 29]. Besides the workplace, interventions implemented in a community setting also appeared to work in reducing blood pressure. A study that included participants age 55 years or more in Asia found that people who attended 60-min Tai Chi three times per week for 12 weeks had a larger reduction in SBP (MD, − 14.30 mmHg; 95% CI, − 19.20 to − 9.40) and in DBP (MD, − 7.02 mmHg; 95% CI, − 10.62 to − 3.42) compared to people maintaining usual daily activities [30]. Another study among patients with hypertension in Asia found that education about the nutritional behavior and guidelines from dietary approaches to stop hypertension (DASH) approach could help reduce blood pressure more in the intervention group compared to the control group who only received the instruction booklets used in intervention group (SBP: MD, − 13.50 mmHg; 95% CI, − 16.15 to − 10.85; DBP: MD, − 6.60 mmHg; 95% CI, − 8.17 to − 5.03) [34]. One study in Africa also showed that education on promoting PA and healthy diet, combined with free smoking cessation consultations could help reduce SBP in the intervention group [25].

##### Management intervention strategies

Two [19, 39] of three [19, 38, 39] (66.7%) studies showed that the management intervention strategies could help reduce mean blood pressure compared to the control group. A study in the US showed that supporting diabetes patients’ self-management of hypertension by team-based chronic models (e.g., proactive patient outreach, depression screening, and health coaching) could decrease more DBP over a 6-month period compared to the usual care (MD, − 1.13 mmHg; 95% CI, − 2.23 to − 0.04) [19]. A study among hypertension patients in Asia showed that improving the social health insurance system by increasing outpatient expenditure reimbursement ratio could help reduce more SBP (MD, − 2.9 mmHg; *P* = 0.01) compared to outpatient expense not covered [38]. The other study among diabetes patients in the US also showed that team-based treatment with trained staff on medical management and self-management helped lower SBP (MD, − 0.88 mmHg; *P* = 0.01), but it did not compare the MD between treatment and control group [39].

##### Education, counseling and management intervention strategies

Six [26, 40, 42–45] of seven [26, 40–45] (85.7%) studies showed that the combination of education, counseling and management intervention strategies led to more blood pressure reduction compared to the control group. One study among hypertension patients in Europe found that management of stress by biofeedback-assisted relaxation and lifestyle counseling on diet and PA reduced more SBP (MD, − 2.62 mmHg; 95% CI, − 3.96 to − 1.29) and DBP (MD, − 1.00 mmHg; 95% CI, − 1.90 to − 0.93) compared to the control group [40]. One study among hypertension patients in the US also found that education about hypertension and home blood pressure monitoring could help reduce more SBP (MD, − 4.70 mmHg; 95% CI, − 7.14 to − 2.26) and DBP (MD, − 2.20 mmHg; 95% CI, − 3.80 to − 0.60) compared to controls [26]. A study among 65-year-and-older hypertension patients in Asia found that the intervention group who received education on hypertension management, community-based eHealth monitoring, and monthly telephone counseling had more reduction in SBP (MD, − 10.80 mmHg; 95% CI, − 14.99 to − 6.61) compared to the control group who only received a poster about hypertension management [42]. A study among hypertension patients in the US also showed that interventions on lifestyle modifications, and nutritional, pharmacological therapies as well as medication adherence lowered SBP and DBP compared to the control group [43]. A study among hypertension patients in Asia found that integration of preventive-curative services delivery and cooperation among village-town-county physicians for education on lifestyle modifications, taking blood pressure drugs regularly and monitoring the blood pressure could help reduce blood pressure more in the intervention group [44]. The other study in Asia also found that integrated program with health education on home blood pressure monitoring and hypertension measurement skills could help reduce blood pressure more in the intervention group [45].

##### Screening and referral for management intervention strategies

Four [16, 46–48] of five [16, 46–49] (80.0%) studies showed that the screening and referral for management intervention strategies could help reduce more blood pressure compared to the control group. Screening for medical or economic needs followed by offering treatment and resources has been found helpful. One study in the US found that screening for unmet needs in primary care and offering those who screened positive some resources could reduce SBP (MD, − 2.6 mmHg; 95% CI, − 3.5 to − 1.7]) and DBP (MD, − 1.4 mmHg; 95% CI, − 1.9 to − 0.9) in patients [16]. The other study among patients with serious mental illness in the US also found that using registry for general medical needs and outcomes, screening and referral for general medical illness prevention and treatment could help reduce more DBP compared to controls (MD, − 3.00 mmHg; 95% CI, − 4.96 to − 1.04) [46]. Assessing and screening CVD risk followed by a management program has also been found beneficial to reduce blood pressure. A study in Europe showed that participating in CVD risk assessment and management program, including screening and tailored strategies for lifestyle advice on CVD risk factors could reduce more SBP (MD, − 2.51 mmHg; 95% CI, − 2.77 to − 2.25) and DBP (MD, − 1.46 mmHg; 95% CI, − 1.62 to − 1.29) compared to controls [47]. A study among hypertension patients in Asia also found that a standardized CVD-risk assessment, a hypertension complication screening and adherence to medications could help reduce more blood pressure compared to the usual care [48].

### Meta-analysis of the effectiveness of interventions on mean blood pressure change

#### Intervention type sub-group analysis

The largest blood pressure reduction (SBP: WMD, − 5.34 mmHg; 95% CI, − 7.35 to − 3.33; DBP: WMD, − 3.23 mmHg; 95% CI, − 5.51 to − 0.96) was seen for interventions that combined education, counseling and management intervention strategies (Table 2**,** Fig. 2).

#### Intervention setting sub-group analysis

Participants who experienced interventions implemented in community settings (WMD, − 3.77 mmHg; 95% CI, − 6.17 to − 1.37) and in health center settings (WMD, − 3.77 mmHg; 95% CI, − 5.78 to − 1.76) had large SBP reduction. Participants experienced interventions implemented in organization settings had large DBP reduction (WMD, − 3.92 mmHg; 95% CI, − 5.80 to − 2.04) (Table 2, Fig. S3).

#### Intervention duration sub-group analysis

Participants who were followed for less than 12 months (i.e., short-term interventions) had a large reduction in blood pressure (SBP: WMD, − 6.25 mmHg; 95% CI, − 9.28 to − 3.21; DBP: WMD, − 3.54 mmHg; 95% CI, − 5.21 to − 1.87) and participants who were followed for longer than or equal to 12 months (i.e., long-term interventions) had a moderate reduction in blood pressure (SBP: WMD, − 1.89 mmHg; 95% CI, − 2.80 to − 0.97; DBP: WMD, − 1.33 mmHg; 95% CI, − 2.11 to − 0.55) (Table 2, Fig. S4).

## Discussion

We summarized the evidence from quasi-experiments that have evaluated interventions used to (1) prevent hypertension in the general population, (2) improve hypertension control in patients with hypertension or (3) reduce blood pressure levels in both the general population and patients.

In this systematic review, we found that the intervention strategies such as (1) education and counseling, (2) management, (3) education, counseling and management and (4) screening and referral for management were beneficial in preventing, controlling hypertension or reducing blood pressure levels. In particular, we found that education and counseling on lifestyle modifications (i.e., promoting PA, healthy diet, smoking cessation consultations) could help prevent hypertension in healthy people. The use of computerized clinical practice guidelines by general practitioners, education and management of hypertension, screening for CVD goals and referral to management could help improve hypertension control in patients with hypertension. The education and counseling on lifestyle modifications, the monitoring of patients’ metabolic factors and chronic diseases (e.g., blood pressure, cholesterol level, etc.) as well as patients’ adherence to medication, the combined education and management of hypertension, the screening for economic risk factors, medical needs, and CVD risk factors, followed by the referral to management all could help reduce blood pressure levels. Our study is one of the few systematic reviews that have summarized the evidence from quasi-experiments on hypertension prevention and control. A previous systematic review [52] which summarized evidence from cluster-randomized trials and quasi-experimental studies had been conducted and found that education, counseling and management strategies were also beneficial in controlling hypertension and reducing blood pressure. It showed that educating healthcare providers and patients, facilitating relay of clinical data to providers, promoting patients’ accesses to resources were associated with improved hypertension control and decreased blood pressure [52]. Another systematic review which summarized evidence from RCTs found that several interventions including blood pressure self-monitoring, educational strategies, improving the delivery of care, and appointment reminder systems could help control hypertension and reduce blood pressure [6]. Another study also found that community-based health workers interventions including health education and counseling, navigating the health care system, managing care, as well as giving social services and support had a significant effect on improving hypertension control and decreasing blood pressure [53]. A review from observational studies and RCT evidence from the US Preventive Services Task Force found that office measurement of blood pressure could effectively screen adults for hypertension [7].

Our review did not find natural experiments studies according to the definition used in this study. Quasi-experimental designs included DID, propensity score matching and pre-post designs with a control group (PPCG). While PPCG designs generally involve two groups (intervention and control) and two different time points (before and after the intervention), DID designs generally involve two or more intervention and control groups and multiple time points [13]. In this review, we did not include pre-post without a control group design because of its higher risk to internal validity due to the absence of comparison to adjust for time trends and confounding [22]. The findings in this review, highlight that, quasi-experiments are increasingly used to evaluate the effectiveness of health interventions for hypertension management when RCTs are not feasible or appropriate. For instance, several studies included in our systematic review often indicated that RCTs would have been difficult to be implemented given that the intervention was conducted in a particular setting such as a pragmatic clinical setting [16, 43, 45, 48], a community setting [24, 35, 36, 42], or a real-world organizational setting [33] because of ethical concerns and human resources issues. Another reason why quasi-experiments were chosen had to do with the need for translation and generalizability of the evidence in a specific community setting [32]. In fact, RCTs are not always generalizable to the communities or settings of interests [11]. The growing interest in and hence the increase in the use of natural and quasi-experiments in public health may be due to the recognition and realization of its usefulness in evaluating health interventions [14, 54].

Given that there was high heterogeneity in the studies included in this systematic review, we have performed a random effects model and have only presented the subgroup analysis by intervention types, settings and duration of the study. Overall, our study suggested that interventions that combined education, counseling and management strategies appeared to show a relatively large beneficial effect for reducing blood pressure. However, our finding should be interpreted with caution due to the high-risk of bias and lower quality of evidence given the quasi-experimental nature of the designs (as opposed to evidence from randomized experiments). Nevertheless, the findings here can give us some insights on the benefit of interventions such as education, counseling and management, especially given that our findings are in line with previous studies [6, 8, 52, 55]. Given that RCTs are not always feasible or appropriate, scientists should develop more rigorous methods to increase the internal validity of non-randomized studies. Compared to previous studies, one systematic review with meta-analysis including cluster-randomized trials and quasi-experiment studies showed that multi-component interventions which incorporated education of health care providers and patients, facilitating relay of clinical data to providers, and promoting patients’ accesses to resources could reduce more blood pressure compared to controls [52]. A recent systematic review with meta-analysis of RCTs also reported that interventions which included blood pressure self-monitoring, appointment reminder systems, educational strategies, and improving the delivery of care showed beneficial effects on lowering blood pressure [6]. Another systematic review and meta-analysis of RCTs also showed that self-measured blood pressure monitoring lowered SBP by 3.9 mmHg and DBP by 2.4 mmHg at 6 months compared to the usual care group [8]. One systematic review and meta-analysis of RCTs found that diet improvement, aerobic exercise, alcohol and sodium restriction, and fish oil supplements reduced blood pressure as well [55].

### Limitations

This review has some limitations. First, the definition of natural and quasi-experiments is not consistent across fields. Second, the main limitation in most if not all the quasi-experimental study designs noted in this review was the potential for unobserved and uncontrolled confounding, which is a threat to internal validity and could lead to biased findings. Third, our findings may not be generalizable to all countries and settings as we only included studies published in the English language in this review. Fourth, as is the case in most other reviews, we could have missed relevant studies despite our best attempt to conduct a thorough search of the literature. Fifth, we found that most studies included in this study had a high risk of bias. It might be because we used the Cochrane Risk of Bias Tool to assess bias which was designed for examining RCTs. Studies in this review only used quasi-experiment designs and did not have randomization, allocation concealment, blinding of participants and personnel, and blinding of outcome assessment. Sixth, studies generally reported the measure of intervention impact differently across studies, making it difficult to combine the findings. In addition, studies were highly heterogeneous in terms of the types of individuals included in the study (e.g., healthy individuals and patients). We conducted the subgroup meta-analysis to reduce the heterogeneity, but the high heterogeneity still existed. Therefore, the results from meta-analysis need to be interpreted with caution. The individual impact reported for each individual study and the results from systematic review should be given more consideration.

## Conclusions

In this systematic review, interventions that used education and counseling strategies; those that used management strategies; those that combined education, counseling and management strategies and those that used screening and referral for management strategies were beneficial in preventing, controlling hypertension and reducing blood pressure levels. The combination of education, counseling and management strategies appeared to be the most beneficial intervention to reduce blood pressure levels. The findings in this review, highlight that, a number of interventions that aim at preventing, controlling hypertension or reducing blood pressure levels are being evaluated through the use of quasi-experimental studies. Given that RCTs are not always feasible or appropriate, scientists should develop more rigorous methods to increase the internal validity of such quasi-experimental studies.

## Supplementary Information

**Additional file 1: Table S1.** Search words. **Table S2.** Summary of the characteristics of the studies included in this review (*n* = 30). **Table S3.** Risk of Bias Tool Assessments Across Studies (n = 30). **Table S4.** GRADE Evidence Profiles Across Studies in Meta-analysis (*n* = 24). **Table S5.** Estimates and parameters in studies that reported on the mean difference in blood pressure (*n* = 27). **Table S6.** Sensitivity analysis for systolic blood pressure (SBP) and diastolic blood pressure (DBP) in meta-analysis stratified by intervention type. **Table S7.** Sensitivity analysis for systolic blood pressure (SBP) and diastolic blood pressure (DBP) in meta-analysis stratified by intervention setting. **Table S8.** Sensitivity analysis for systolic blood pressure (SBP) and diastolic blood pressure (DBP) in meta-analysis stratified by intervention duration. **Fig. S1.** Methods to calculate mean differences (MD). **Fig. S2.** Methods to calculate standard errors (SE). **Fig. S3.** Forest plot stratified by intervention settings for blood pressure. **(A)** Forest plot stratified by intervention settings for systolic blood pressure (SBP). **(B)** Forest plot stratified by intervention settings for diastolic blood pressure (DBP). **Fig. S4.** Forest plot stratified by intervention duration for blood pressure. (**A)** Forest plot stratified by intervention duration for systolic blood pressure (SBP). (**B)** Forest plot stratified by intervention duration for diastolic blood pressure (DBP). **Fig. S5.** Funnel plot of systolic blood pressure (SBP), diastolic blood pressure (DBP) stratified by intervention types. **Fig. S6.** Funnel plot of systolic blood pressure (SBP), diastolic blood pressure (DBP) stratified by intervention settings. **Fig. S7.** Funnel plot of systolic blood pressure (SBP), diastolic blood pressure (DBP) stratified by intervention duration. **Fig. S8.** Sensitivity analysis of systolic blood pressure (SBP), diastolic blood pressure (DBP) stratified by intervention types. **Fig. S9.** Sensitivity analysis of systolic blood pressure (SBP), diastolic blood pressure (DBP) stratified by intervention settings. **Fig. S10.** Sensitivity analysis of systolic blood pressure (SBP), diastolic blood pressure (DBP) stratified by intervention duration

## Data Availability

The data supporting the conclusions of this article is included within the article and the additional file.

## Abbreviations

CI: Confidence interval
CVD: Cardiovascular disease
DASH: Dietary approaches to stop hypertension
DBP: Diastolic blood pressure
DID: Difference-in-difference
GRADE: Grading of Recommendations, Assessment, Development, and Evaluation
MD: Mean difference
PA: Physical activity
PPCG: Pre-post designs with a control group
RCT: Randomized clinical trial
SBP: Systolic blood pressure
SE: Standard error
US: United States
WMD: Weighted mean difference
