# Supplementary material for: Interventions in hypertension: systematic review and meta-analysis of natural and quasi-experiments
Source: Clin Hypertens. 2022 May 1;28:13. doi: 10.1186/s40885-022-00198-2 (PMC9057066; doi:10.1186/s40885-022-00198-2)
Supplement: Supplementary file 1 — Additional file 1: Table S1. Search words. Table S2. Summary of the characteristics of the studies included in this review (n = 30). Table S3. Risk of Bias Tool Assessments Across Studies (n = 30). Table S4. GRADE Evidence Profiles Across Studies in Meta-analysis (n = 24). Table S5. Estimates and parameters in studies that reported on the mean difference in blood pressure (n = 27). Table S6. Sensitivity analysis for systolic blood pressure (SBP) and diastolic blood pressure (DBP) in meta-analysis stratified by intervention type. Table S7. Sensitivity analysis for systolic blood pressure (SBP) and diastolic blood pressure (DBP) in meta-analysis stratified by intervention setting. Table S8. Sensitivity analysis for systolic blood pressure (SBP) and diastolic blood pressure (DBP) in meta-analysis stratified by intervention duration. Fig. S1. Methods to calculate mean differences (MD). Fig. S2. Methods to calculate standard errors (SE). Fig. S3. Forest plot stratified by intervention settings for blood pressure. (A) Forest plot stratified by intervention settings for systolic blood pressure (SBP). (B) Forest plot stratified by intervention settings for diastolic blood pressure (DBP). Fig. S4. Forest plot stratified by intervention duration for blood pressure. (A) Forest plot stratified by intervention duration for systolic blood pressure (SBP). (B) Forest plot stratified by intervention duration for diastolic blood pressure (DBP). Fig. S5. Funnel plot of systolic blood pressure (SBP), diastolic blood pressure (DBP) stratified by intervention types. Fig. S6. Funnel plot of systolic blood pressure (SBP), diastolic blood pressure (DBP) stratified by intervention settings. Fig. S7. Funnel plot of systolic blood pressure (SBP), diastolic blood pressure (DBP) stratified by intervention duration. Fig. S8. Sensitivity analysis of systolic blood pressure (SBP), diastolic blood pressure (DBP) stratified by intervention types. Fig. S9. Sensitivity analysis of systolic blood pressu [file 40885_2022_198_MOESM1_ESM.docx]

**Supplemental file**

**Title: Interventions in Hypertension: Systematic Review and Meta-analysis of Natural and Quasi-Experiments**

Table of Contents

[Table S1. Search words 3](#_Toc90721729)

[Table S2. Summary of the characteristics of the studies included in this review (n = 30) 4](#_Toc90721730)

[Table S3. Risk of Bias Tool Assessments Across Studies (n = 30) 5](#_Toc90721731)

[Table S4. GRADE Evidence Profiles Across Studies in Meta-analysis (n = 24) 6](#_Toc90721732)

[Table S5. Estimates and parameters in studies that reported on the mean difference in blood pressure (n = 27) 7](#_Toc90721733)

[Table S6. Sensitivity analysis for systolic blood pressure (SBP) and diastolic blood pressure (DBP) in meta-analysis stratified by intervention type 9](#_Toc90721734)

[Table S7. Sensitivity analysis for systolic blood pressure (SBP) and diastolic blood pressure (DBP) in meta-analysis stratified by intervention setting 10](#_Toc90721735)

[Table S8. Sensitivity analysis for systolic blood pressure (SBP) and diastolic blood pressure (DBP) in meta-analysis stratified by intervention duration 11](#_Toc90721736)

[Fig. S1. Methods to calculate mean differences (MDs) 13](#_Toc90721737)

[Fig. S2. Methods to calculate standard errors (SEs) 14](#_Toc90721738)

[Fig. S3. Forest plot stratified by intervention settings for blood pressure 15](#_Toc90721739)

[(A). Forest plot stratified by intervention settings for systolic blood pressure (SBP) 15](#_Toc90721740)

[(B). Forest plot stratified by intervention settings for diastolic blood pressure (DBP) 16](#_Toc90721741)

[Fig. S4. Forest plot stratified by intervention duration for blood pressure 17](#_Toc90721742)

[(A). Forest plot stratified by intervention duration for systolic blood pressure (SBP) 17](#_Toc90721743)

[(B). Forest plot stratified by intervention duration for diastolic blood pressure (DBP) 18](#_Toc90721744)

[Fig. S5. Funnel plot of systolic blood pressure (SBP), diastolic blood pressure (DBP) stratified by intervention types 19](#_Toc90721745)

[Fig. S6. Funnel plot of systolic blood pressure (SBP), diastolic blood pressure (DBP) stratified by intervention settings 20](#_Toc90721746)

[Fig. S7. Funnel plot of systolic blood pressure (SBP), diastolic blood pressure (DBP) stratified by intervention duration 21](#_Toc90721747)

[Fig. S8. Sensitivity analysis of systolic blood pressure (SBP), diastolic blood pressure (DBP) stratified by intervention types 22](#_Toc90721748)

[Fig. S9. Sensitivity analysis of systolic blood pressure (SBP), diastolic blood pressure (DBP) stratified by intervention settings 23](#_Toc90721749)

[Fig. S10. Sensitivity analysis of systolic blood pressure (SBP), diastolic blood pressure (DBP) stratified by intervention duration 24](#_Toc90721750)

# **Table S1.** Search words

| **Database** | **Search words** |
| --- | --- |
| Pubmed | English[Language] AND ("hypertension"[All fields] OR "blood pressure"[All fields] OR "hypertensive"[All fields] OR hyperten*[All fields] OR "high blood pressure"[All fields] OR "elevated blood pressure"[All fields] OR " abnormal blood pressure"[All fields] ) AND ("intervention"[all fields] OR "program"[all fields] OR "policy"[all fields] OR "policies"[all fields]) AND ("2008/01/01”[PDAT] : "2018/11/13”[PDAT]) AND ("environment"[all fields] OR "neighborhood"[all fields] OR "school"[all fields] OR "workplace"[all fields] OR "community"[all fields] OR " environmental"[all fields]) AND ("natural experiment"[all fields]OR "natural experiments"[all fields]OR "quasi-experiment"[all fields] OR "quasi-experimental"[all fields]OR " synthetic control method"[all fields] OR " difference-in-difference"[all fields] OR " instrumental variables"[all fields] OR " propensity score"[all fields] OR " interrupted time series"[all fields] OR " regression discontinuity"[all fields] OR "prepost"[all fields] OR "pre post"[all fields]) |
| Web of science: choose topic | ("hypertension" OR "blood pressure" OR "hypertensive" OR hyperten* OR "high blood pressure" OR "elevated blood pressure" OR " abnormal blood pressure" ) AND ("intervention" OR "program" OR "policy" OR "policies") AND ("environment" OR "neighborhood" OR "school" OR "workplace" OR "community" OR " environmental") AND ("natural experiment" OR "natural experiments"OR "quasi-experiment" OR "quasi-experimental"OR " synthetic control method" OR " difference-in-difference" OR " instrumental variables" OR " propensity score" OR " interrupted time series" OR " regression discontinuity" OR "prepost" OR "pre post") |
| Embase | 'hypertension'/exp OR 'hypertension':ti,ab,kw OR 'blood pressure':ti,ab,kw OR 'hypertensive':ti,ab,kw OR 'hyperten*':ti,ab,kw OR 'high blood pressure':ti,ab,kw OR 'elevated blood pressure':ti,ab,kw OR 'abnormal blood pressure':ti,ab,kw  AND 'health care policy'/exp OR 'intervention':ti,ab,kw OR 'program':ti,ab,kw OR 'policy':ti,ab,kw OR 'policies':ti,ab,kw  AND 'school'/exp OR 'environment':ti,ab,kw OR 'neighborhood':ti,ab,kw OR 'school':ti,ab,kw OR 'workplace':ti,ab,kw OR 'community':ti,ab,kw OR 'environmental':ti,ab,kw  AND 'quasi experimental study'/exp OR 'propensity score'/exp OR 'natural experiment':ti,ab,kw OR 'natural experiments':ti,ab,kw OR 'quasi-experiment':ti,ab,kw OR 'quasi-experimental':ti,ab,kw OR 'synthetic control method':ti,ab,kw OR 'difference-in-difference':ti,ab,kw OR 'instrumental variables':ti,ab,kw OR 'propensity score':ti,ab,kw OR 'interrupted time series':ti,ab,kw OR 'regression discontinuity':ti,ab,kw OR 'prepost':ti,ab,kw OR 'pre post':ti,ab,kw  AND (2008:py OR 2009:py OR 2010:py OR 2011:py OR 2012:py OR 2013:py OR 2014:py OR 2015:py OR 2016:py OR 2017:py OR 2018:py)  AND [english]/lim |

# **Table S2.** Summary of the characteristics of the studies included in this review (n = 30)

| **Study characteristics** | **Studies**  **n (%)** |
| --- | --- |
| **Study Design** |  |
| Pre-post design with a control group (PPCG) | 16 (53.33) |
| Difference-in-difference (DID) | 10 (33.33) |
| Propensity-score-matched (PSM) designs | 1 (3.33) |
| DID & PSM | 3 (10.00) |
| **Geographic Region** |  |
| Europe | 5 (16.67) |
| Africa | 2 (6.67) |
| America | 12 (40.00) |
| Asia | 11 (36.67) |
| **Specific subpopulations included** |  |
| *Based on gender* |  |
| Including only women | 2 (6.67) |
| Including both genders | 28 (93.33) |
| *Based on race/ethnicity* |  |
| Including only Black/African American, Latino or Hispanic | 1 (3.33) |
| Not specific | 29 (96.67) |
| **Data Source** |  |
| Primary data | 25 (83.33) |
| Secondary data | 2 (6.67) |
| Primary and Secondary | 3 (10.00) |
| **Type of Intervention** |  |
| Education and counseling | 13 (43.33) |
| Management | 4 (13.33) |
| Education, counseling and management | 7 (23.33) |
| Screening and referral for management | 6 (20.00) |
| **Level/Setting of Intervention** |  |
| Organizational level | 4 (13.33) |
| Health center level | 12 (40.00) |
| Nation level | 2 (6.67) |
| Community level | 12 (40.00) |
| **Intervention duration** |  |
| Short duration | 14 (46.7) |
| Long duration | 16 (53.3) |
| **Outcomes reported** |  |
| Changes in mean systolic and diastolic blood pressure only | 25 (83.33) |
| Changes in hypertension prevalence only | 3 (10.00) |
| Changes in mean systolic and diastolic blood pressure and hypertension prevalence | 2 (6.67) |

PPCG, pre-post design with a control group; DID, difference-in-difference; PSM design, propensity-score-matched design

# **Table S3.** Risk of Bias Tool Assessments Across Studies (n = 30)

|  | **Random Sequence Generation** | **Allocation Concealment** | **Blinding of Participants & Personnel** | **Blinding of Outcome Assessment** | **Incomplete Outcome Data** | **Selecting Reporting** | **Other Sources of Bias** |
| --- | --- | --- | --- | --- | --- | --- | --- |
| Barnidge, 2015 | High risk | High risk | High risk | High risk | Low risk | Low risk | Low risk |
| Berkowitz, 2017 | High risk | High risk | High risk | High risk | Low risk | Low risk | Low risk |
| Chang, 2013 | High risk | High risk | High risk | High risk | Low risk | Low risk | Low risk |
| Chang, 2016 | High risk | High risk | Low risk | Low risk | Low risk | Low risk | Low risk |
| Comin, 2017 | High risk | High risk | Low risk | Low risk | Low risk | Low risk | Low risk |
| Darviri, 2016 | High risk | High risk | High risk | High risk | Low risk | Low risk | Low risk |
| Fernandez, 2008 | High risk | High risk | High risk | High risk | Low risk | Low risk | Low risk |
| Flannery, 2012 | Low risk | High risk | High risk | High risk | Low risk | Low risk | Low risk |
| Gemson, 2008 | High risk | High risk | High risk | High risk | Low risk | Low risk | Low risk |
| Hussain, 2016 | High risk | High risk | High risk | High risk | Low risk | Low risk | Low risk |
| James, 2018 | High risk | High risk | Low risk | Low risk | Low risk | Low risk | Low risk |
| Jung and Lee, 2017 | High risk | High risk | High risk | High risk | Low risk | Low risk | Low risk |
| Lin, 2017 | Low risk | High risk | High risk | High risk | Low risk | Low risk | Low risk |
| Fikri-Benbrahim, 2012 | Low risk | Low risk | Low risk | Low risk | Low risk | Low risk | Low risk |
| Panattoni, 2017 | High risk | High risk | High risk | High risk | Low risk | Low risk | Low risk |
| Scanlon, 2008 | High risk | High risk | High risk | High risk | Low risk | Low risk | Low risk |
| Scharf, 2016 | High risk | High risk | High risk | High risk | Low risk | Low risk | Low risk |
| Verberne, 2016 | High risk | High risk | Low risk | Low risk | Low risk | Low risk | Low risk |
| Xu, 2015 | High risk | High risk | High risk | High risk | Low risk | Low risk | Low risk |
| Yu, 2017 | High risk | High risk | High risk | High risk | Low risk | Low risk | Low  risk |
| Kamran, 2016 | Low risk | Low risk | High risk | High risk | Low risk | Low risk | Low risk |
| Ibrahim, 2016 | High risk | High risk | High risk | High risk | Low risk | Low risk | Low risk |
| Kassim, 2017 | High risk | High risk | High risk | High risk | Low risk | Low risk | Low risk |
| Fazliana, 2018 | High risk | High risk | High risk | High risk | Low risk | Low risk | Low risk |
| Sahli, 2016 | High risk | High risk | High risk | High risk | Low risk | Low risk | Low risk |
| Miao, 2016 | High risk | High risk | High risk | High risk | Low risk | Low risk | Low risk |
| Miao, 2018 | High risk | High risk | High risk | High risk | Low risk | Low risk | Low risk |
| Visanuyothin,, 2018 | Low risk | Low risk | High risk | Low risk | Low risk | Low risk | Low risk |
| van de Vijver, 2016 | High risk | High risk | High risk | High risk | Low risk | Low risk | Low risk |
| Zhu, 2018 | High risk | High risk | High risk | High risk | Low risk | Low risk | Low risk |

# **Table S4.** GRADE Evidence Profiles Across Studies in Meta-analysis (n = 24)

| **Outcomes** | **Risk of Bias^1^** | **Inconsistency^2^** | **Indirectness^3^** | **Imprecision^4^** | **Publication Bias^5^** | **Magnitude of Effect^6^** | **Residual^7^** | **Dose-Response^8^** | **Overall GRADE Quality** |
| --- | --- | --- | --- | --- | --- | --- | --- | --- | --- |
| Mean SBP Change | Low (remain) | Very low (down 1) | Moderate (remain) | High (remain) | Very low (down 2) | High (remain) | Low (remain) | Low (remain) | Low |
| Mean DBP Change | Low (remain) | Very low (down 1) | Moderate (remain) | High (remain) | Very low (down 2) | High (remain) | Low (remain) | Low (remain) | Low |

^1^ Most studies in each outcome had significant limitations in the study design (high risk of most items in Appendix Table 3).

^2^ The I^2^ statistic indicated heterogeneity, 91.2% and 91.8% respectively.

^3^ We only included studies published in the English language.

^4^ Large sample size for power analysis for the estimates tested on the outcomes.

^5^ Funnel plots showed that the presence of publication bias was very likely.

^6^ The mean SBP and DBP outcomes showed large significant effects.

^7^ Data about confounders were available in most studies.

^8^ Lack of evidence in dose-response relationship between interventions and outcomes.

GRADE: Grading of Recommendations, Assessment, Development, and Evaluation; SBP: systolic blood pressure; DBP: diastolic blood pressure.

# **Table S5.** Estimates and parameters in studies that reported on the mean difference in blood pressure (n = 27)

| **First author, year** | **Findings** | **First author, year** | **Findings** | **First author, year** | **Findings** |
| --- | --- | --- | --- | --- | --- |
| Berkowitz, 2017 | SBP_MD = –2.6;  SBP_SE = 0.46  DBP_MD = –1.4  DBP_SE = 0.26  MD_Source = 1  SE_Source = 2 | Chang, 2013 | SBP_MD = –14.3;  SBP_SE = 2.5  DBP_MD = –7.02  DBP_SE = 1.84  MD_Source = 1  SE_Source = 2 | Verberne, 2016 | SBP_MD = –0.5;  SBP_SE = 1.12  DBP_MD = 0.2  DBP_SE = 0.63  MD_Source = 2  SE_Source = 5 |
| Chang, 2016 | SBP_MD = –2.51;  SBP_SE = 0.13  DBP_MD = –1.46  DBP_SE = 0.08  MD_Source = 1  SE_Source = 2 | Fernandez, 2008 | SBP_MD = –2.4;  SBP_SE = 3.76  DBP_MD = –2.6  DBP_SE = 1.98  MD_Source = 2  SE_Source = 4 | Xu, 2015 | SBP_MD = –8.9;  SBP_SE = 5.23  DBP_MD = –3.4  DBP_SE = 3.29  MD_Source = 1  SE_Source = 2 |
| Darviri, 2016 | SBP_MD = –2.62;  SBP_SE = 0.68  DBP_MD = –1  DBP_SE = 0.25  MD_Source = 1  SE_Source = 2 | Fikri-Benbrahim, 2012 | SBP_MD = –4.7;  SBP_SE = 1.24  DBP_MD = –2.2  DBP_SE = 0.82  MD_Source = 2  SE_Source = 4 | Panattoni, 2017 | SBP_MD = –1;  SBP_SE = 0.47  DBP_MD = –0.7  DBP_SE = 0.28  MD_Source = 2  SE_Source = 5 |
| Flannery, 2012 | SBP_MD = –4.44;  SBP_SE = 5.31  DBP_MD = –6.7  DBP_SE = 3.39  MD_Source = 2  SE_Source = 5 | Gemson, 2008 | SBP_MD = –3.2;  SBP_SE = 1.14  DBP_MD = –3  DBP_SE = 1.36  MD_Source = 2  SE_Source = 4 | Scharf, 2016 | SBP_MD = –1;  SBP_SE = 1.64  DBP_MD = –3  DBP_SE = 1  MD_Source = 2  SE_Source = 6 |
| Hussain, 2016 | SBP_MD = 9;  DBP_MD = 4  MD_Source = 1 | Jung and Lee, 2017 | SBP_MD = –10.8;  SBP_SE = 2.14  DBP_MD = –3.6  DBP_SE = 1.59  MD_Source = 2  SE_Source =4 | Yu, 2017 | SBP_MD = –0.85;  SBP_SE = 0.18  DBP_MD = 0.02  DBP_SE = 0.17  MD_Source = 2  SE_Source = 5 |
| Scanlon, 2008 | BaselineSBP > 140_MD_per year  = -2.2;  BaselineSBP > 140_MD_per year_SE = 0.88;  BaselineSBP > 140_MD_per year_Source = 1;  BaselineSBP > 140_MD_per year_SE_Source = 2 | Lin, 2017 | SBP_MD = –2.1;  SBP_SE = 2.02  DBP_MD = –5.2  DBP_SE = 1.48  MD_Source = 2  SE_Source = 5 | Zhu, 2018 | SBP_MD = –2.7 ;  SBP_SE = 5.06  DBP_MD = –0.1  DBP_SE = 3.41  MD_Source = 2  SE_Source = 5 |
| Kamran, 2016 | SBP_MD = –13.5;  SBP_SE = 1.35  DBP_MD = –6.6  DBP_SE = 0.80  MD_Source = 2  SE_Source = 4 | Ibrahim, 2016 | SBP_MD = –1.71;  SBP_SE = 1.16  DBP_MD = –2.63  DBP_SE = 0.59  MD_Source = 1  SE_Source = 2 | Kassim, 2017 | SBP_MD = –0.2;  SBP_SE = 1.69  DBP_MD = –0.28  DBP_SE = 1.17  MD_Source = 2  SE_Source = 5 |
| Visanuyothin, 2018 | SBP_MD = –7.13;  SBP_SE = 2.28  DBP_MD = –4.66  DBP_SE = 1.35  MD_Source = 2  SE_Source = 5 | Miao, 2018 | SBP_MD = –2.9;  DBP_MD = –7.9  MD_Source = 1 | Miao, 2016 | SBP_MD = –5.62;  SBP_SE = 0.44  DBP_MD = –5.43  DBP_SE = 0.39  MD_Source = 1  SE_Source =1 |
| van de Vijver, 2016 | SBP_MD = –0.32;  SBP_SE = 1.10  DBP_MD = 1.09  DBP_SE = 0.70  MD_Source = 1  SE_Source = 2 | Fazliana, 2018 | SBP_MD = 1.14;  SBP_SE = 1.14  DBP_MD = 0.02  DBP_SE = 0.75  MD_Source = 2  SE_Source = 2&5 | Sahli, 2016 | SBP_MD = –2.5;  SBP_SE = 0.81  DBP_MD = –0.4  DBP_SE = 0.50  MD_Source = 2  SE_Source = 5 |

SBP, systolic blood pressure; DBP, diastolic blood pressure; MD, mean difference; SE, standard error; Source refers to the methods used to calculate the mean difference and the standard error.

# **Table S6.** Sensitivity analysis for systolic blood pressure (SBP) and diastolic blood pressure (DBP) in meta-analysis stratified by intervention type

| **Study omitted** | **Mean SBP change** | | | **Mean DBP change** | | |
| --- | --- | --- | --- | --- | --- | --- |
|  | **Estimate** | **95% Confidence interval** | | **Estimate** | **95% Confidence interval** | |
|  |  | **Lower limit** | **Upper limit** |  | **Lower limit** | **Upper limit** |
| **Education and counseling** |  |  |  |  |  |  |
| Flannery, 2012 | –4.06 | –6.90 | –1.22 | –2.48 | –4.08 | –0.88 |
| Gemson, 2008 | –4.21 | –7.33 | –1.09 | –2.62 | –4.31 | –0.93 |
| Lin, 2017 | –4.28 | –7.24 | –1.32 | –2.40 | –4.03 | –0.77 |
| Chang, 2013 | –3.14 | –5.78 | –0.51 | –2.29 | –3.87 | –0.70 |
| Verberne, 2016 | –4.50 | –7.55 | –1.44 | –3.00 | –4.71 | –1.29 |
| Xu, 2015 | –3.85 | –6.68 | –1.03 | –2.61 | –4.24 | –0.99 |
| Zhu, 2018 | –4.14 | –6.98 | –1.30 | –2.75 | –4.37 | –1.13 |
| Kamran, 2016 | –2.63 | –4.54 | –0.73 | –1.96 | –3.23 | –0.69 |
| Ibrahim, 2016 | –4.37 | –7.47 | –1.27 | –2.69 | –4.52 | –0.86 |
| Kassim, 2017 | –4.48 | –7.45 | –1.52 | –2.90 | –4.60 | –1.20 |
| Fazliana, 2018 | –4.66 | –7.57 | –1.75 | –2.97 | –4.70 | –1.25 |
| Sahli, 2016 | –4.32 | –7.61 | –1.03 | –2.96 | –4.76 | –1.15 |
| Combined | –4.07 | –6.83 | –1.32 | –2.64 | –4.22 | –1.06 |
| **Education, counseling and management** |  |  |  |  |  |  |
| Darviri, 2016 | –6.09 | –7.91 | –4.28 | –3.87 | –5.58 | –2.15 |
| Fernandez, 2008 | –5.54 | –7.65 | –3.43 | –3.32 | –5.80 | –0.85 |
| Fikri-Benbrahim, 2012 | –5.57 | –8.04 | –3.11 | –3.46 | –6.18 | –0.74 |
| Jung and Lee, 2017 | –4.54 | –6.39 | –2.70 | –3.17 | –5.68 | –0.66 |
| Miao, 2016 | –5.43 | –8.35 | –2.52 | –2.42 | –3.87 | –0.96 |
| Visanuyothin, 2018 | –5.12 | –7.31 | –2.93 | –2.96 | –5.49 | –0.44 |
| Combined | –5.34 | –7.35 | –3.33 | –3.23 | –5.51 | –0.96 |
| **Screening and referral for management** |  |  |  |  |  |  |
| Berkowitz, 2017 | –1.35 | –2.69 | –0.02 | –0.71 | –1.90 | 0.48 |
| Scharf, 2016 | –1.72 | –2.88 | –0.55 | –0.58 | –1.54 | 0.38 |
| Chang, 2016 | –1.35 | –2.57 | –0.12 | –0.68 | –1.86 | 0.50 |
| Yu, 2017 | –2.31 | –2.95 | –1.66 | –1.17 | –1.93 | –0.41 |
| van de Vijver, 2016 | –1.87 | –3.06 | –0.68 | –1.21 | –2.16 | –0.27 |
| Combined | –1.66 | –2.77 | –0.55 | –0.86 | –1.76 | 0.05 |

SBP, systolic blood pressure; DBP, diastolic blood pressure.

# **Table S7.** Sensitivity analysis for systolic blood pressure (SBP) and diastolic blood pressure (DBP) in meta-analysis stratified by intervention setting

| **Study omitted** | **Mean SBP change** | | | **Mean DBP change** | | |
| --- | --- | --- | --- | --- | --- | --- |
|  | **Estimate** | **95% Confidence interval** | | **Estimate** | **95% Confidence interval** | |
|  |  | **Lower limit** | **Upper limit** |  | **Lower limit** | **Upper limit** |
| **Community** |  |  |  |  |  |  |
| Chang, 2013 | –2.61 | –4.62 | –0.60 | –1.14 | –2.23 | –0.05 |
| Xu, 2015 | –3.56 | –6.01 | –1.12 | –1.53 | –2.77 | –0.28 |
| Ibrahim, 2016 | –4.14 | –6.92 | –1.37 | –1.39 | –2.67 | –0.10 |
| Kassim, 2017 | –4.24 | –6.86 | –1.62 | –1.75 | –3.07 | –0.42 |
| Fazliana, 2018 | –4.44 | –6.96 | –1.91 | –1.84 | –3.21 | –0.48 |
| Sahli, 2016 | –4.11 | –7.07 | –1.15 | –1.84 | –3.30 | –0.39 |
| Fernandez, 2008 | –3.88 | –6.39 | –1.36 | –1.52 | –2.79 | –0.25 |
| Fikri-Benbrahim, 2012 | –3.70 | –6.36 | –1.03 | –1.52 | –2.87 | –0.17 |
| Jung and Lee, 2017 | –2.90 | –5.14 | –0.67 | –1.40 | –2.66 | –0.15 |
| van de Vijver, 2016 | –4.31 | –7.02 | –1.60 | –1.92 | –3.12 | –0.72 |
| Combined | –3.77 | –6.17 | –1.37 | –1.58 | –2.79 | –0.36 |
| **Health center** |  |  |  |  |  |  |
| Verberne, 2016 | –4.24 | –6.43 | –2.05 | –2.97 | –4.62 | –1.33 |
| Kamran, 2016 | –2.44 | –4.15 | –0.72 | –2.00 | –3.46 | –0.53 |
| Panattoni, 2017 | –4.27 | –6.72 | –1.82 | –2.89 | –4.76 | –1.02 |
| Miao, 2016 | –3.37 | –5.28 | –1.47 | –1.98 | –3.14 | –0.82 |
| Visanuyothin, 2018 | –3.46 | –5.54 | –1.37 | –2.34 | –3.92 | –0.77 |
| Berkowitz, 2017 | –4.01 | –6.47 | –1.56 | –2.79 | –4.72 | –0.86 |
| Scharf, 2016 | –4.10 | –6.25 | –1.96 | –2.51 | –4.12 | –0.91 |
| Yu, 2017 | –4.31 | –6.77 | –1.84 | –3.00 | –4.75 | –1.24 |
| Combined | –3.77 | –5.78 | –1.76 | –2.57 | –4.07 | –1.06 |
| **Nation** |  |  |  |  |  |  |
| Darviri, 2016 | –2.51 | –2.77 | –2.25 | –1.46 | –1.62 | –1.30 |
| Chang, 2016 | –2.62 | –3.95 | –1.29 | –1.00 | –1.48 | –0.52 |
| Combined | –2.51 | –2.77 | –2.26 | –1.29 | –1.72 | –0.85 |
| **Organization** |  |  |  |  |  |  |
| Flannery, 2012 | –2.92 | –4.84 | –1.01 | –3.65 | –5.80 | –1.49 |
| Gemson, 2008 | –2.43 | –5.89 | 1.03 | –4.60 | –7.46 | –1.74 |
| Lin, 2017 | –3.23 | –5.37 | –1.09 | –3.10 | –5.42 | –0.78 |
| Zhu, 2018 | –2.98 | –4.90 | –1.07 | –4.22 | –6.11 | –2.34 |
| Combined | –2.97 | –4.86 | –1.09 | –3.92 | –5.80 | –2.04 |

SBP, systolic blood pressure; DBP, diastolic blood pressure.

# **Table S8.** Sensitivity analysis for systolic blood pressure (SBP) and diastolic blood pressure (DBP) in meta-analysis stratified by intervention duration

| **Study omitted** | **Mean SBP change** | | | **Mean DBP change** | | |
| --- | --- | --- | --- | --- | --- | --- |
|  | **Estimate** | **95% Confidence interval** | | **Estimate** | **95% Confidence interval** | |
|  |  | **Lower limit** | **Upper limit** |  | **Lower limit** | **Upper limit** |
| **Short duration** |  |  |  |  |  |  |
| Flannery, 2012 | –6.34 | –9.49 | –3.20 | –3.40 | –5.11 | –1.70 |
| Lin, 2017 | –6.68 | –9.96 | –3.39 | –3.38 | –5.12 | –1.63 |
| Chang, 2013 | –5.49 | –8.49 | –2.48 | –3.24 | –4.93 | –1.55 |
| Xu, 2015 | –6.11 | –9.25 | –2.97 | –3.55 | –5.28 | –1.82 |
| Zhu, 2018 | –6.44 | –9.59 | –3.29 | –3.69 | –5.42 | –1.97 |
| Kamran, 2016 | –5.31 | –7.80 | –2.83 | –3.00 | –4.38 | –1.62 |
| Kassim, 2017 | –6.91 | –10.14 | –3.69 | –3.91 | –5.74 | –2.08 |
| Darviri, 2016 | –6.68 | –10.09 | –3.27 | –3.90 | –5.51 | –2.29 |
| Fernandez, 2008 | –6.53 | –9.71 | –3.34 | –3.62 | –5.39 | –1.85 |
| Fikri-Benbrahim, 2012 | –6.42 | –9.95 | –2.88 | –3.72 | –5.68 | –1.77 |
| Jung and Lee, 2017 | –5.78 | –8.95 | –2.62 | –3.54 | –5.33 | –1.76 |
| Visanuyothin, 2018 | –6.16 | –9.43 | –2.89 | –3.43 | –5.20 | –1.65 |
| Combined | –6.25 | –9.28 | –3.21 | –3.54 | –5.21 | –1.87 |
| **Long duration** |  |  |  |  |  |  |
| Gemson, 2008 | –1.79 | –2.74 | –0.84 | –1.25 | –2.05 | –0.45 |
| Verberne, 2016 | –1.99 | –2.94 | –1.04 | –1.46 | –2.28 | –0.65 |
| Ibrahim, 2016 | –1.90 | –2.85 | –0.94 | –1.21 | –2.03 | –0.39 |
| Fazliana, 2018 | –2.11 | –3.04 | –1.18 | –1.44 | –2.26 | –0.62 |
| Sahli, 2016 | –1.83 | –2.79 | –0.86 | –1.42 | –2.25 | –0.59 |
| Panattoni, 2017 | –1.98 | –2.96 | –0.99 | –1.40 | –2.27 | –0.53 |
| Miao, 2016 | –1.50 | –2.27 | –0.73 | –0.87 | –1.47 | –0.28 |
| Berkowitz, 2017 | –1.79 | –2.80 | –0.79 | –1.33 | –2.22 | –0.44 |
| Scharf, 2016 | –1.93 | –2.87 | –0.99 | –1.22 | –2.03 | –0.41 |
| Chang, 2016 | –1.75 | –2.98 | –0.52 | –1.33 | –2.41 | –0.25 |
| Yu, 2017 | –2.01 | –3.00 | –1.02 | –1.48 | –2.32 | –0.64 |
| van de Vijver, 2016 | –2.00 | –2.95 | –1.05 | –1.53 | –2.34 | –0.73 |
| Combined | –1.89 | –2.80 | –0.97 | –1.33 | –2.11 | –0.55 |

SBP, systolic blood pressure; DBP, diastolic blood pressure.

# **Fig. S1.** Methods to calculate mean differences (MDs)

DID, difference-in-difference; MD, mean difference.

# **Fig. S2.** Methods to calculate standard errors (SEs)

SE, standard error; SD, standard deviation; CI, confidence interval; UL, upper level; LL, lower level.

# **Fig. S3.** Forest plot stratified by intervention settings for blood pressure

## **(A).** Forest plot stratified by intervention settings for systolic blood pressure (SBP)

SBP, systolic blood pressure; HBP, high blood pressure.

## **(B).** Forest plot stratified by intervention settings for diastolic blood pressure (DBP)

DBP, diastolic blood pressure; HBP, high blood pressure.

# **Fig. S4.** Forest plot stratified by intervention duration for blood pressure

## **(A).** Forest plot stratified by intervention duration for systolic blood pressure (SBP)

SBP, systolic blood pressure; HBP, high blood pressure.

## **(B).** Forest plot stratified by intervention duration for diastolic blood pressure (DBP)

DBP, diastolic blood pressure; HBP, high blood pressure.


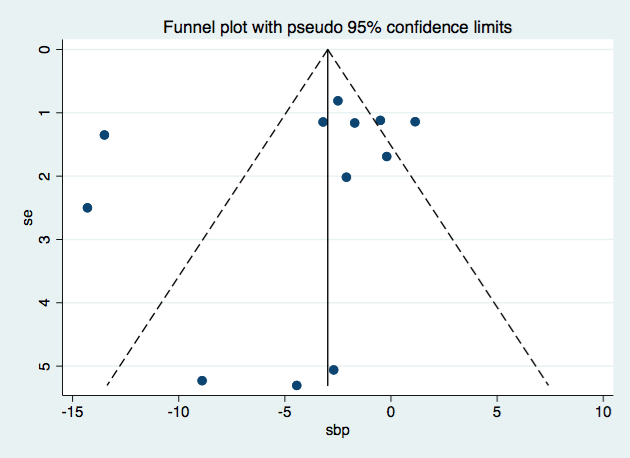

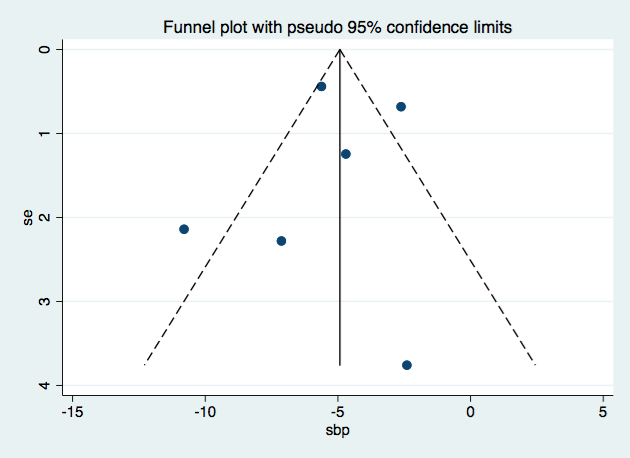

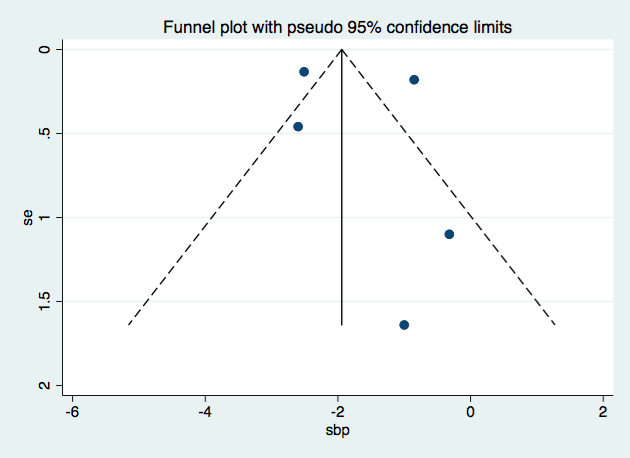


**(A).** Education and counseling for SBP **(B).** Education, counseling and management for SBP **(C).** Screening, referral and management for SBP


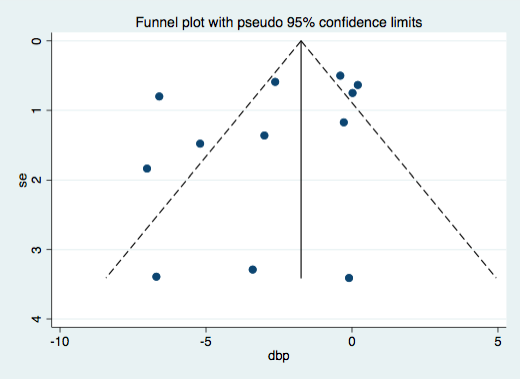

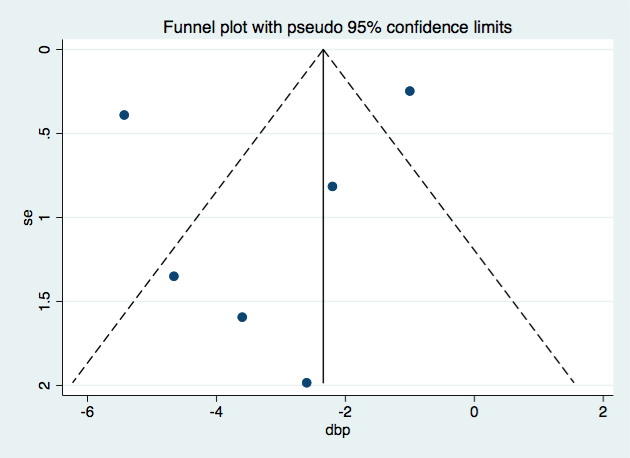

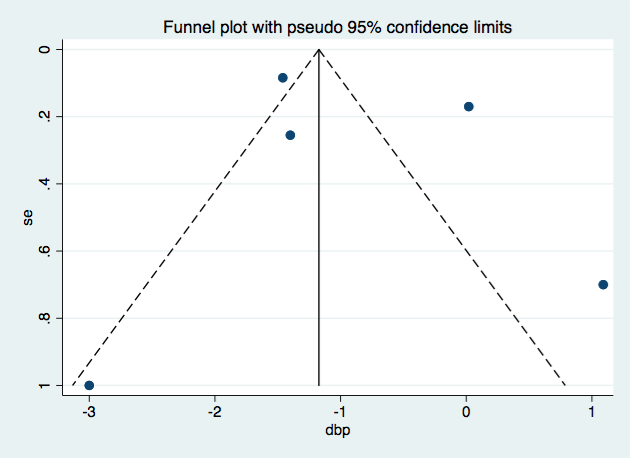


**(D).** Education and counseling for DBP **(E).** Education, counseling and management for DBP **(F).** Screening, referral and management for DBP

# **Fig. S5.** Funnel plot of systolic blood pressure (SBP), diastolic blood pressure (DBP) stratified by intervention types

SBP, systolic blood pressure; DBP, diastolic blood pressure.


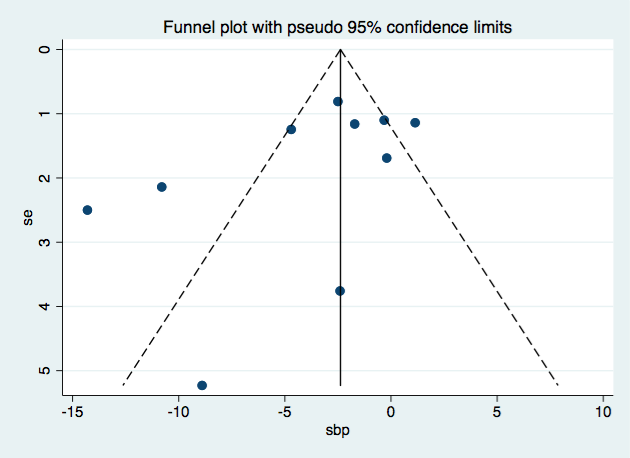

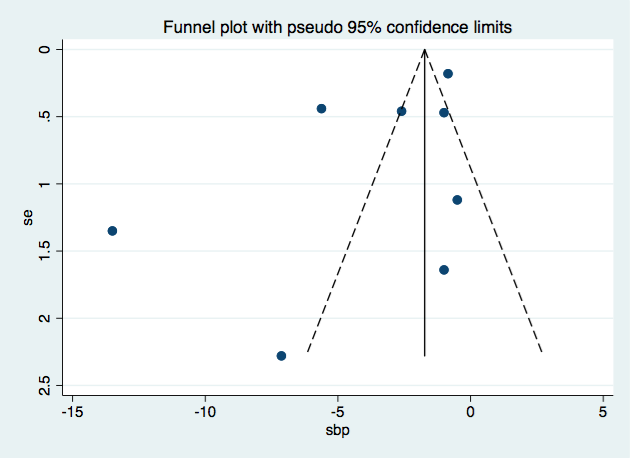

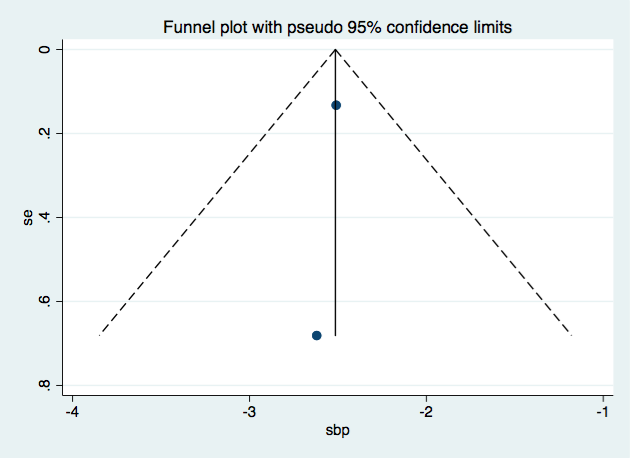

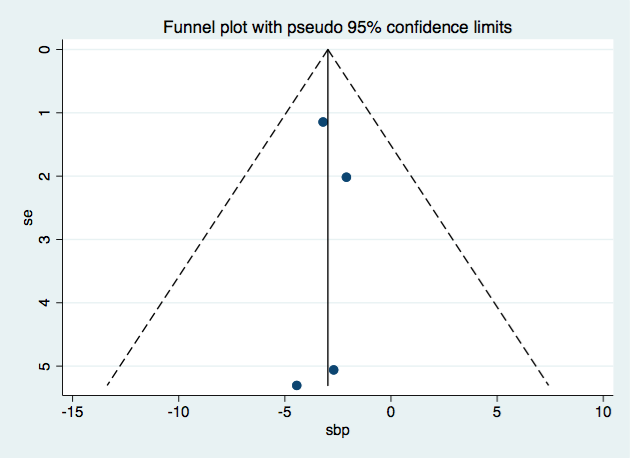


**(A).** Community setting for SBP **(B).** Health center setting for SBP **(C).** Nation setting for SBP **(D).** Organization setting for SBP


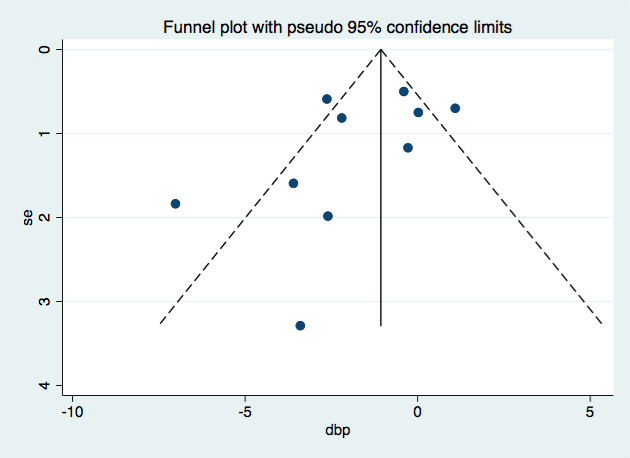

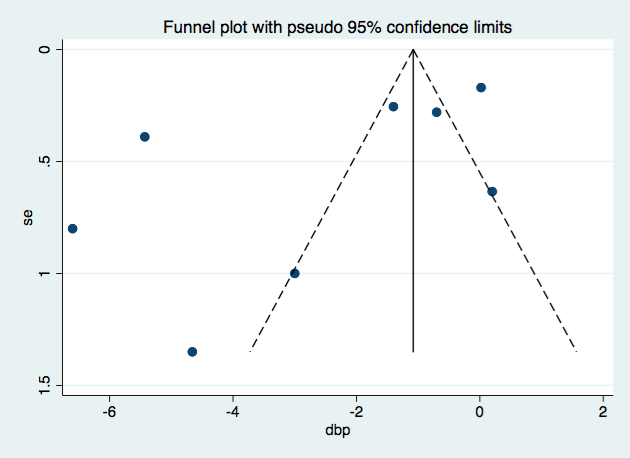

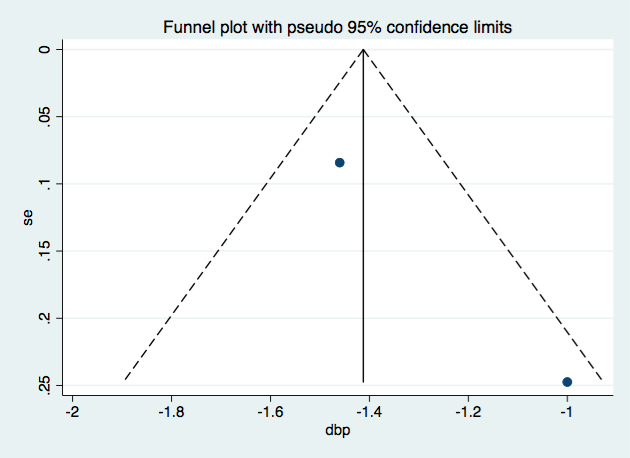

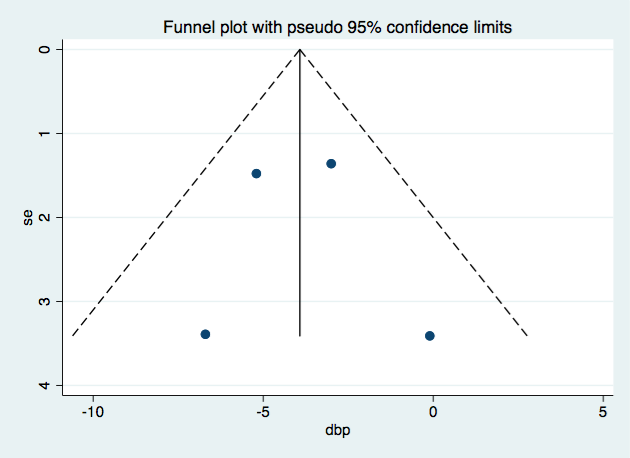


**(E).** Community setting for DBP **(F).** Health center setting for DBP **(G).** Nation setting for DBP **(H).** Organization setting for DBP

# **Fig. S6.** Funnel plot of systolic blood pressure (SBP), diastolic blood pressure (DBP) stratified by intervention settings

SBP, systolic blood pressure; DBP, diastolic blood pressure.


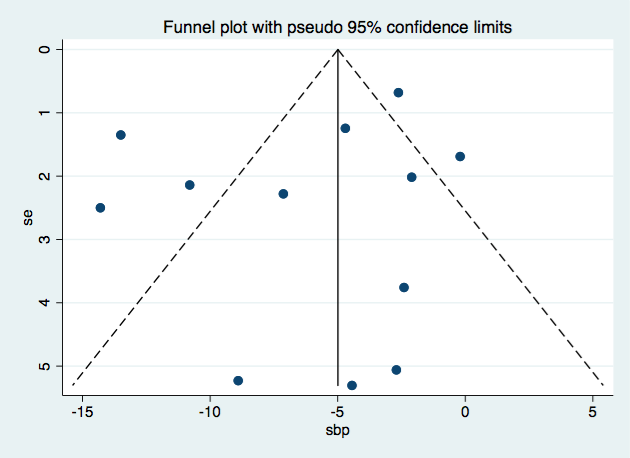

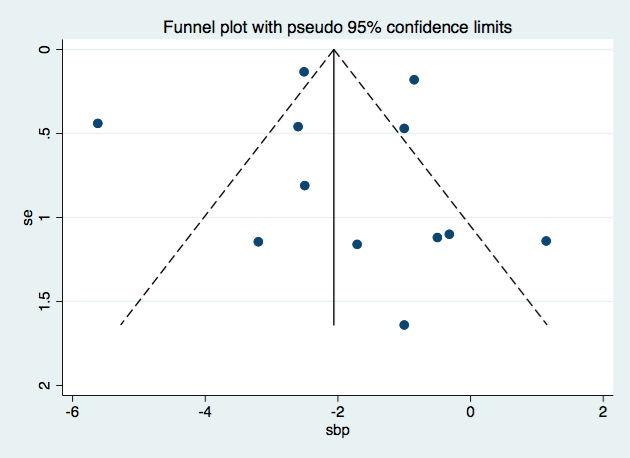

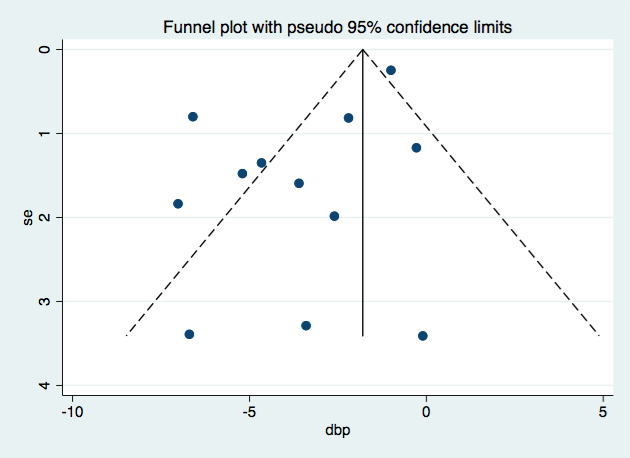

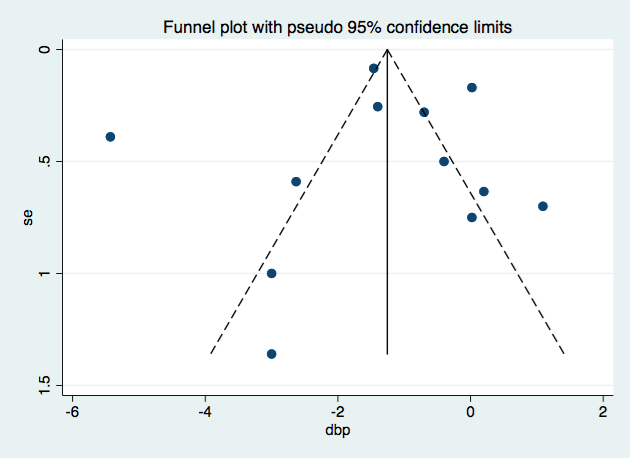


**(A).** Short duration for SBP **(B).** Long duration for SBP **(C).** Short duration for DBP **(D).** Long duration for DBP

# **Fig. S7.** Funnel plot of systolic blood pressure (SBP), diastolic blood pressure (DBP) stratified by intervention duration

SBP, systolic blood pressure; DBP, diastolic blood pressure.


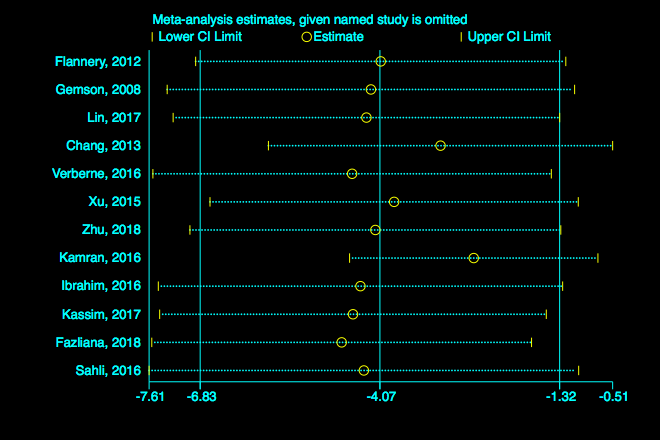

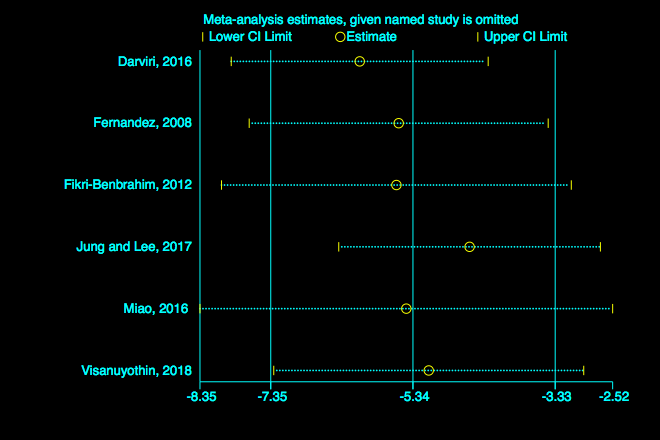

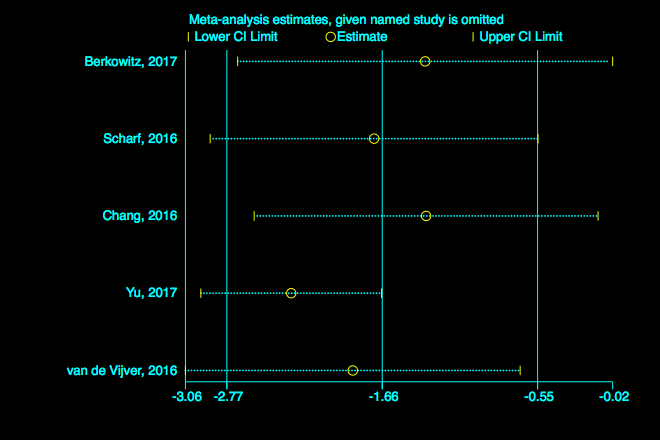


**(A).** Education and counseling for SBP **(B).** Education, counseling and management for SBP **(C).** Screening, referral and management for SBP
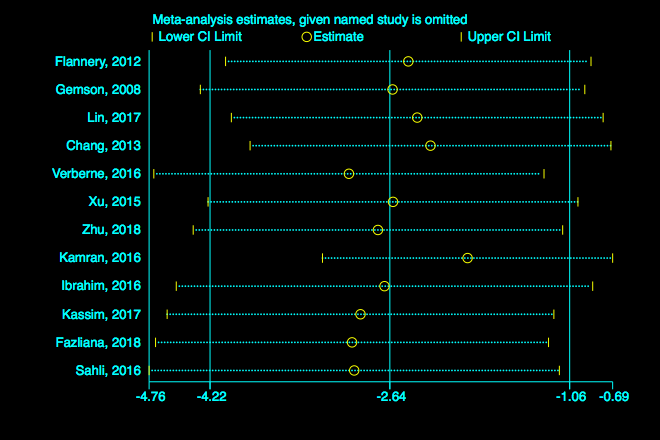

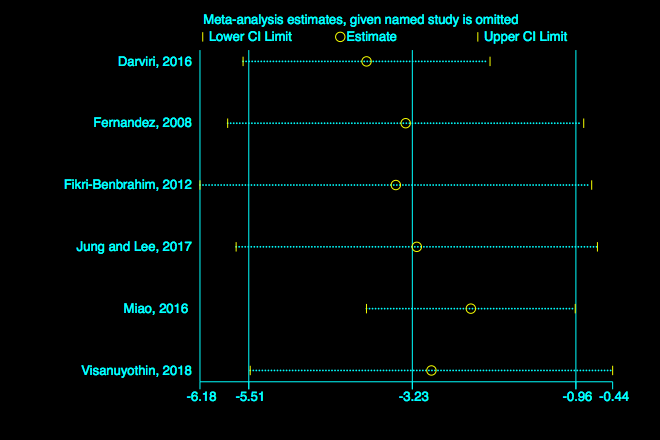

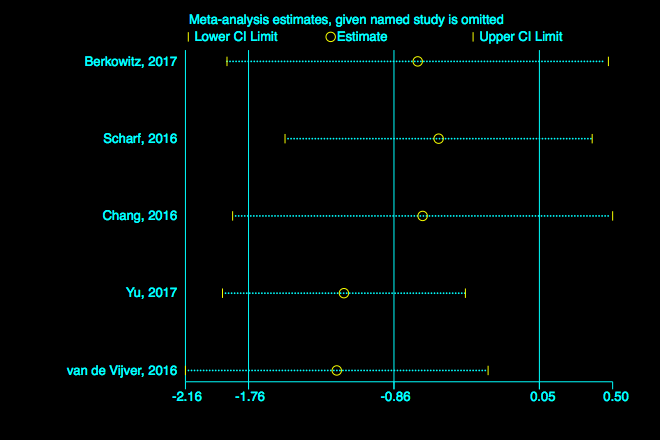


**(D).** Education and counseling for DBP **(E).** Education, counseling and management for DBP **(F).** Screening, referral and management for DBP

# **Fig. S8.** Sensitivity analysis of systolic blood pressure (SBP), diastolic blood pressure (DBP) stratified by intervention types

SBP, systolic blood pressure; DBP, diastolic blood pressure.


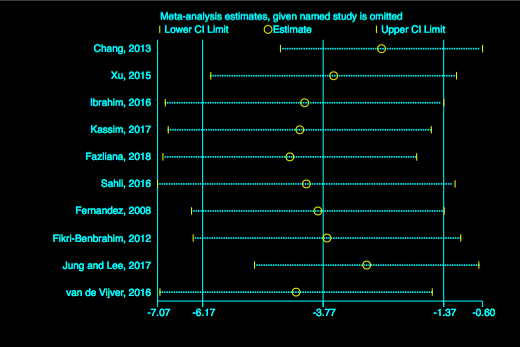

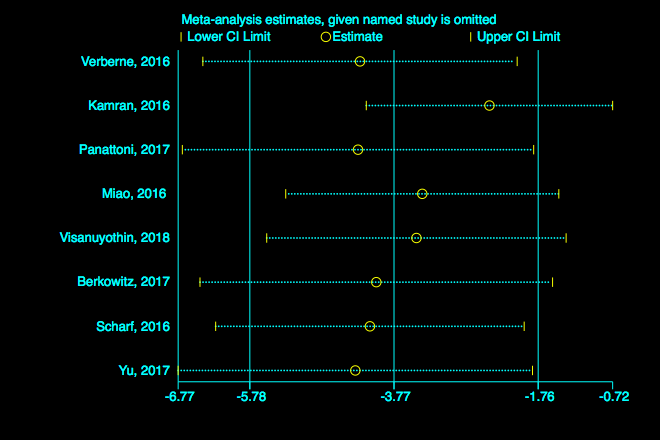

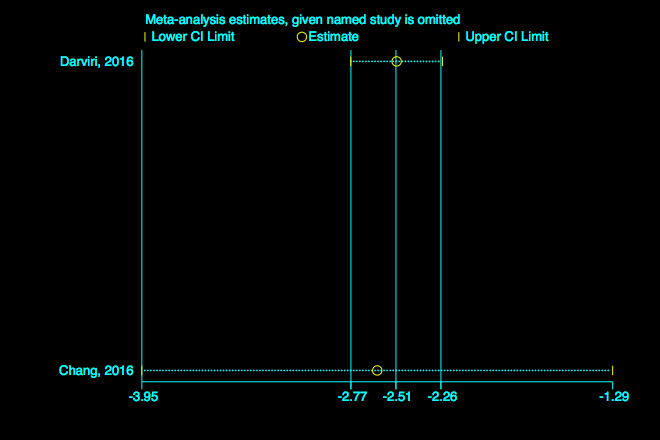

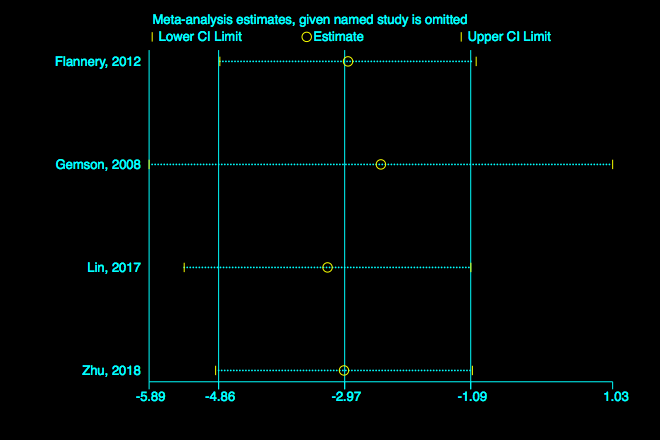


**(A).** Community setting for SBP **(B).** Health center setting for SBP **(C).** Nation setting for SBP **(D).** Organization setting for SBP


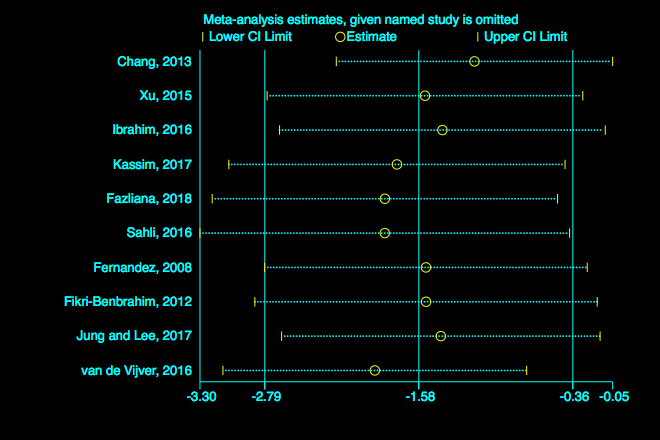

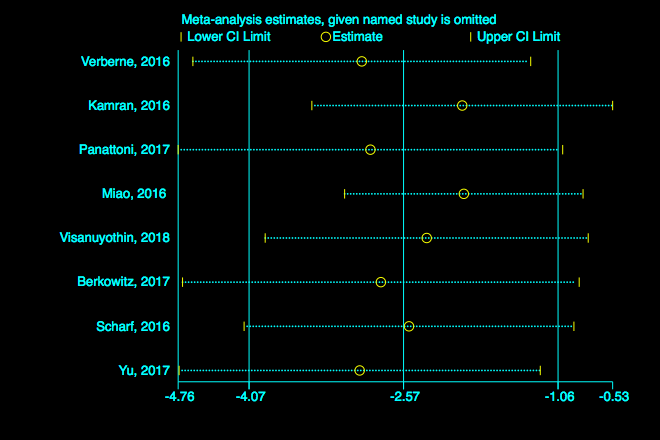

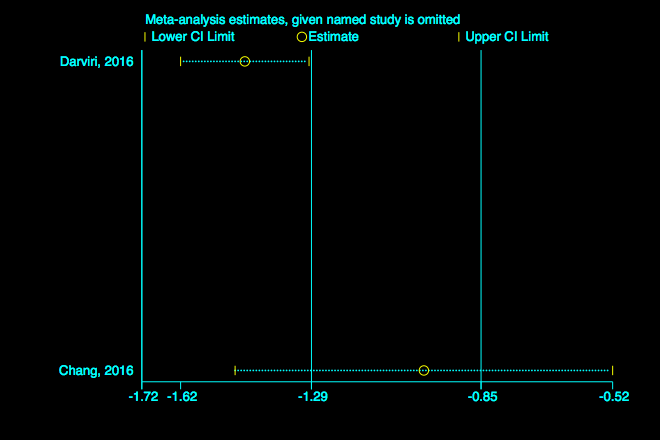

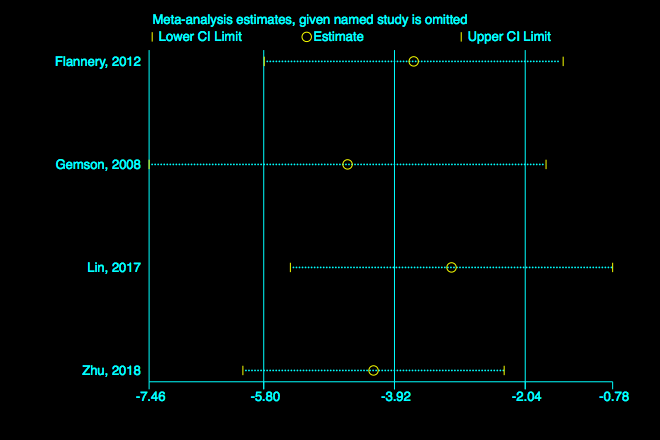


**(E).** Community setting for DBP **(F).** Health center setting for DBP **(G).** Nation setting for DBP **(H).** Organization setting for DBP

# **Fig. S9.** Sensitivity analysis of systolic blood pressure (SBP), diastolic blood pressure (DBP) stratified by intervention settings

SBP, systolic blood pressure; DBP, diastolic blood pressure.


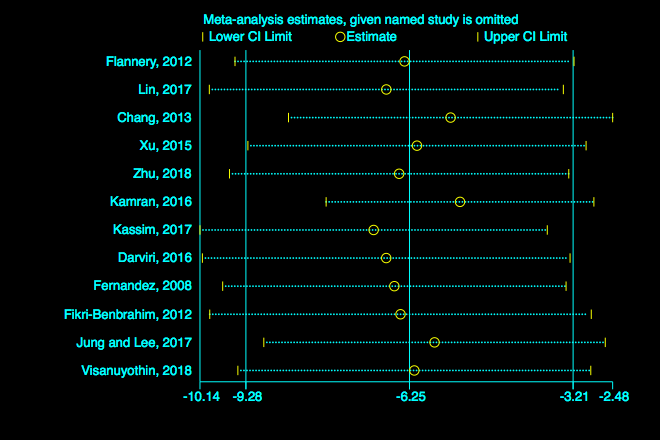

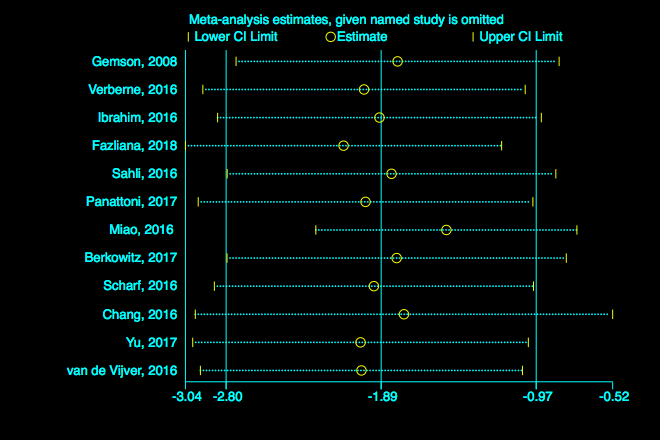

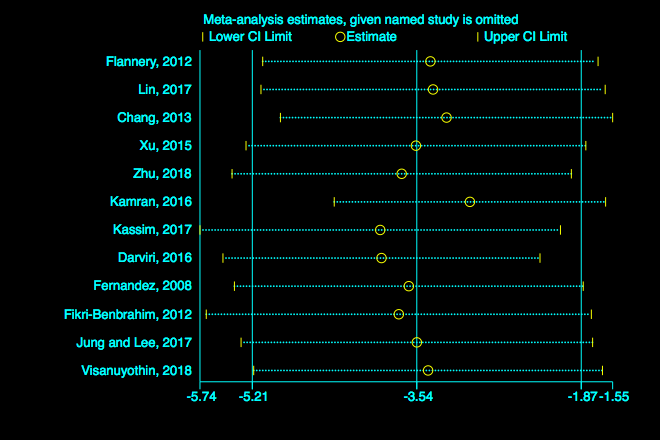

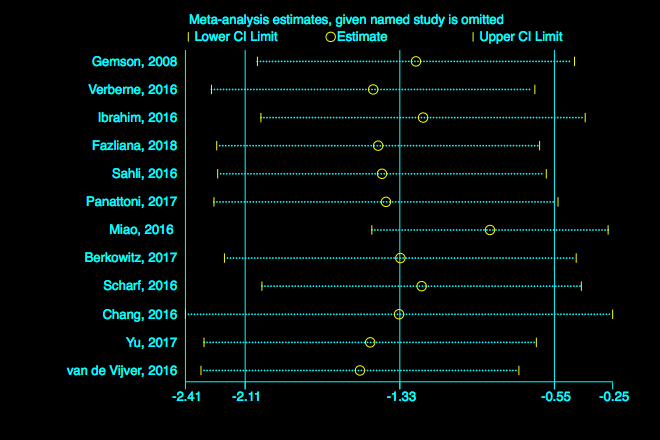


**(A).** Short duration for SBP **(B).** Long duration for SBP **(C).** Short duration for DBP **(D).** Long duration for DBP

# **Fig. S10.** Sensitivity analysis of systolic blood pressure (SBP), diastolic blood pressure (DBP) stratified by intervention duration

SBP, systolic blood pressure; DBP, diastolic blood pressure.
